# A Web-Based Lifestyle-Related Course for People Living With Multiple Sclerosis: Quantitative Evaluation of Course Completion, Satisfaction, and Lifestyle Changes Among Participants Enrolled in a Randomized Controlled Trial

**DOI:** 10.2196/59363

**Published:** 2025-05-26

**Authors:** Maggie Yu, Sandra Neate, Steve Simpson-Yap, Rebekah Davenport, William Bevens, George Jelinek, Jeanette Reece

**Affiliations:** 1 Centre for Epidemiology and Biostatistics, Melbourne School of Population and Global Health The University of Melbourne Melbourne Australia; 2 Florey Institute of Neuroscience and Mental Health The University of Melbourne Melbourne Australia; 3 MS Research Flagship, Menzies Institute for Medical Research University of Tasmania Hobart Australia; 4 Melbourne School of Psychological Sciences The University of Melbourne Melbourne Australia; 5 Department of Psychiatry, University of California, San Diego IN STEP Children’s Mental Health Research Center Child & Adolescent Services Research Center (CASRC) La Jolla, CA United States

**Keywords:** multiple sclerosis, randomized controlled trial, lifestyle, multimodal, digital intervention, Multiple Sclerosis Online Course, course engagement

## Abstract

**Background:**

Web-based health courses providing lifestyle-related information can potentially increase knowledge, facilitate behavior change, and improve health outcomes for people living with multiple sclerosis (MS). Despite the low engagement with web-based programs by this population, few studies have evaluated factors influencing engagement. This study evaluated engagement with our 6-week lifestyle-related course (Multiple Sclerosis Online Course; MSOC) by participants enrolled in a large, international randomized controlled trial, as well as preliminary outcomes.

**Objective:**

This study aimed to quantitatively assess engagement with the MSOC (the intervention course [IC] and standard-care course [SCC]), motivators of and barriers to participants’ course completion, course satisfaction, engagement with the community forum, and intentions to implement lifestyle changes.

**Methods:**

We collected data via a baseline survey before course commencement and an evaluation survey 1 month after the 6-week course. Course completers were queried on motivators of completion, course satisfaction, previous knowledge, forum participation, and intentions to adopt lifestyle changes. Noncompleters were queried on barriers to course completion. Differences between the 2 study arms were examined using chi-square and 2-tailed *t* tests. Multivariable linear regression models assessed factors (sociodemographic and course and health related) associated with participants’ intentions to adopt lifestyle changes adjusting for baseline lifestyle factors. Moderation analyses were conducted to test group differences.

**Results:**

Of the 857 participants, 442 (51.6%) completed the MSOC (IC: n=218, 49.3%; SCC: n=224, 50.7%), and 291 (34%) completed the evaluation survey (n=254, 87.3% course completers; n=37, 12.7% noncompleters). Key motivators of course completion included an interest in participating in MS research, optimizing health, course flexibility, and relevant and useful course content. Barriers to course completion included time constraints and technical issues. Most course completers rated the MSOC as “excellent/very good” (IC: 92/126, 73%; SCC: 78/128, 60.9%; *P=*.17). Engagement with the facilitator-led community forum was higher in the IC than in the SCC (56/126, 44.4% vs 32/128, 25%; *P*=.003). More IC completers versus SCC completers expressed their intention to adopt dietary changes (89/125, 71.2% vs 74/127, 58.3%; *P*=.04), increase their sun exposure (82/124, 66.1% vs 62/124, 50%; *P*=.01), supplement with omega-3 (84/125, 67.2% vs 60/126, 47.6%; *P*=.004), and practice meditation (85/124, 68.5% vs 66/126, 52.4%; *P*=.009). Forum engagement, course satisfaction, new course content, and an interest in receiving additional course content were associated with intentions to adopt lifestyle changes across both study arms.

**Conclusions:**

The web-based lifestyle IC provided new and satisfactory content and facilitated intentions to adopt lifestyle changes. Positive associations between engagement with the community forum and intentions to implement lifestyle changes and identifying barriers to completion such as time constraints provide important insights to inform the design of future digital health interventions for people living with MS and possibly other chronic conditions.

**Trial Registration:**

Australian New Zealand Clinical Trials Registry (ANZCTR) ACTRN12621001605886; https://www.anzctr.org.au/Trial/Registration/TrialReview.aspx?id=382778&isReview=true

## Introduction

### Background

Multiple sclerosis (MS) is a chronic inflammatory disease of the central nervous system that affects almost 3 million people worldwide [1]. It is the most common cause of nontraumatic disability in young and middle-aged adults [2] and manifests with symptoms such as fatigue, depression, and anxiety, as well as reduced quality of life (QoL) [3,4].

Although treatments for MS such as disease-modifying therapies (DMTs) can ameliorate disease progression and reduce the risk of relapse (transient exacerbations of new or recurring neurological dysfunction) [5], people living with MS have expressed a strong interest in self-management strategies [6]. Self-management strategies, such as modification of lifestyle-related risk factors (eg, diet, exercise, and stress management), have been shown to be beneficial in managing MS-related symptoms and improving individuals’ sense of control over their disease as an adjunct to pharmacological treatments [7-10]. More recently, web-based interventions have demonstrated effectiveness in promoting self-management strategies to improve MS-related knowledge, self-efficacy, and symptom management [11,12]. Web-based health interventions, particularly lifestyle-related ones, could improve accessibility issues and reduce barriers of geography and mobility, thereby increasing knowledge translation to people living with MS [12-15].

Despite the potential advantages of web-based health interventions for people living with MS in terms of availability and cost-effectiveness, these interventions have encountered issues related to high attrition [16,17] and low engagement rates [18]. Previous studies evaluating participation in web-based health-related courses for people living with MS have also focused primarily on course satisfaction [18], knowledge gain [18], and self-management of fatigue [11,12]. Few studies have comprehensively examined engagement (particularly course commencement and completion) with web-based health interventions to facilitate self-management strategies for people living with MS, including whether knowledge gain through interventions translates into changes in attitudes and lifestyle.

In addition, the influence of social support and interaction on the completion of self-paced web-based interventions for people living with MS remains unclear. While chat forums, online message groups, and web-based coaches have been found to increase course satisfaction [19], there is a noticeable gap in the literature regarding social engagement and the learning outcomes of web-based lifestyle interventions. Given the rapid shift toward web-based health care platforms and their potential benefits for people living with MS, studies exploring engagement with web-based interventions by populations with chronic illnesses such as MS remain a high priority.

We developed the Multiple Sclerosis Online Course (MSOC), a web-based program delivering evidence-based lifestyle recommendations for people living with MS with the aim of improving health and well-being outcomes in this population. After confirming the feasibility, acceptability, and learnability of the MSOC [14], a large randomized controlled trial (RCT) was designed and is currently being conducted to investigate the effectiveness of a multimodal lifestyle intervention course (IC) in improving health outcomes and QoL in people living with MS compared to a standard-care course (SCC) [15].

### Objectives

This study aimed to evaluate engagement with the web-based lifestyle program, the MSOC, by RCT participants (an international cohort of people living with MS) and the impact of the MSOC in facilitating intentions to adopt lifestyle changes *after* participants were provided with access to the MSOC (IC or SCC; Textbox 1). This study used data collected from an evaluation survey 1 month after the delivery of the MSOC and specifically aimed to determine motivators of course completion, levels of course satisfaction, engagement with a facilitator-led community forum, and barriers to engagement among course noncompleters (Multimedia Appendix 1). In addition, we examined intentions to adopt lifestyle changes across the IC and SCC.

**A comparison of aspects of engagement with the Multiple Sclerosis Online Course across the intervention course and standard-care course in course completers and noncompleters.**

**Aspects examined among course completers**

Motivators of course completion

Course satisfaction

Previous knowledge of course content

Participation in the facilitator-run community forum

Intention to adopt recommended lifestyle modifications

Sociodemographic and course- and health-related factors associated with participants’ intention to adopt recommended lifestyle modifications

**Aspects examined among course noncompleters**

Barriers to course completion

Notably, this study complements our previously published quantitative study from the same RCT, which specifically aimed to determine sociodemographic, clinical, and lifestyle-related factors associated with course commencement and completion using participant baseline data [20]. That is, baseline data were collected before participants had access to the MSOC, whereas this study used data collected 1 month after the 6-week course. In another 2 *qualitative* studies from the same RCT, we examined how people living with MS (N=38 course completers) seek information online [21] and participants’ perceptions of their MS and their perceived sense of control over their disease [22].

Overall, this study provides unique insights into factors influencing engagement and lifestyle change intentions and examines the role of course design elements such as facilitator-led forums. The study findings can be used to improve the design of future digital health interventions for people living with MS and potentially other chronic conditions.

## Methods

### Overview

In this ongoing RCT, the IC, based largely on information from the Overcoming MS lifestyle program [23], is being tested for its effectiveness in improving health outcomes in a large international cohort of people living with MS compared to the SCC, which provides general lifestyle recommendations from reputable MS websites, as described fully in the protocol paper [24] (Multimedia Appendices 2 and 3).

Enrolled participants (people living with MS recruited online from the United Kingdom and Ireland, North America, and Australia and New Zealand) were allocated to 1 of 2 courses using simple randomization: the IC or the SCC. The web-based courses were freely delivered worldwide in English via a laptop or PC connected to the internet. Course content was presented in videos, animations, text, and images and structured into seven modules covering the following topics: (1) introduction, (2) eating well (diet recommendations), (3) sunlight and vitamin D, (4) exercise, (5) meditation and the mind-body connection, (6) medication and family prevention, and (7) conclusion (Table S1 in Multimedia Appendix 4). Briefly, the 7 learning modules were delivered biweekly over 4 weeks followed by a 2-week catch-up period (total course length was 6 weeks). Each course also included a facilitator-led community forum.

In the feasibility RCT, while the MSOC was found to be both acceptable and useful by people living with MS, 45% of participants did not complete the course, and there was no engagement with the community forum [25]. As a result, facilitators and researchers were added to the community forum in the MSOC effectiveness RCT [24] to promote dialogue by introducing each module topic, promoting discussion within the module by asking questions related to the topic, and answering participants’ questions with the intent of increasing engagement and, subsequently, course completion.

In total, 5 rounds of recruitment took place via international MS websites and social media: August 1, 2022, to September 12, 2022; October 19, 2022, to November 21, 2022; November 21, 2022, to December 31, 2022; March 27, 2023, to May 7, 2023; and July 24, 2023, to September 3, 2023.

### Ethical Considerations

All participants provided informed consent for their data to be used for research. This study was approved by the University of Melbourne Human Research Ethics Subcommittee (ID: 22140). The trial was registered with the Australian New Zealand Clinical Trials Registry (ACTRN12621001605886). All methods were carried out in accordance with relevant guidelines and regulations. Participants were provided with a detailed plain-language statement explaining the study objectives, procedures, potential risks, and benefits. This study was administered and conducted solely by the Neuroepidemiology Unit at the Centre for Epidemiology and Biostatistics, Melbourne School of Population and Global Health, the University of Melbourne. The single-site nature of this study ensured that all participant data were securely stored in 1 location, on the University of Melbourne server. Participants did not receive monetary compensation for their involvement.

### MSOC Completion

Course completion was defined as completing modules 1 to 6 of the MSOC as module 7 comprised a closing session and did not provide lifestyle-related information. Course noncompletion was defined as completing module 1 but failing to complete module 6. At the end of each run of the 6-week IC and SCC, all participants (including those who did not complete the course) were emailed a voluntary postcourse evaluation survey to complete. Invited participants who did not complete the voluntary evaluation survey were then sent a further email reminder 2 weeks later.

### Participant Sociodemographic and Health Characteristics

Participants submitted a baseline survey before course commencement via the online course platform. The baseline survey collected a range of sociodemographic characteristics (eg, sex, age, highest attained educational level, employment status, partnership status, and country of residence) and health data, including MS type (categorized into nonprogressive [benign or relapsing-remitting MS] and progressive [primary progressive, secondary progressive, or progressive-relapsing MS]); MS duration (in years), derived from the year of diagnosis and the year of survey completion; BMI, calculated from weight and height squared and categorized into underweight (<18.5 kg/m^2^), normal weight (18.5-24.9 kg/m^2^), overweight (25.0-29.9 kg/m^2^), and obese (≥30.0 kg/m^2^) according to World Health Organization guidelines; treated comorbidities queried via the Self-Administered Comorbidity Questionnaire and then dichotomized (≤1 or >1); DMT use (no or yes); and self-reported ongoing symptoms of relapse in the preceding 30 days (no or yes). Participants were also queried about whether they were taking medications for their MS.

### Patient-Reported Outcome Measures

Fatigue was measured using the 9-item Fatigue Severity Scale, with mean Fatigue Severity Scale scores of >5 indicating clinically significant fatigue [26]. Disability was assessed using the Patient-Determined Disease Steps [27,28], a self-reported assessment of ambulatory disability that has been validated against the Expanded Disability Status Scale and is classified into 3 categories: normal or mild (0-2), moderate (3-5), and severe (6-8). The 14-item Hospital Anxiety and Depression Scale was used to assess anxiety and depressive symptoms [29,30]. This tool comprises 7 questions each for anxiety and depression rated on a Likert scale from 0 to 3 and has been validated in people living with MS [31]. Health-related QoL was assessed using the psychometrically validated Multiple Sclerosis Quality of Life–54 (MSQOL-54) instrument [32]. The Physical Health Composite and Mental Health Composite were derived from relevant subscales per the MSQOL-54 guidelines. Although minimal clinically important differences have not been established for the MSQOL-54 composite scores, differences of ≥5 points have previously been determined as the minimum clinically meaningful change in a health-related QoL measure [33,34].

### Motivators of and Barriers to Course Completion

Participants completing either the IC or SCC (completers) were queried on motivating factors that facilitated course completion, with multiple-choice options offered: “Convenience/flexible time and location,” “Opportunity to participate in MS research,” “Topics that were relevant and/or important to me,” “The course was interesting,” “Connection and interaction with other people with MS,” “Engagement with facilitators,” “To optimize my health,” and “Other (specify in text).” Participants could select more than one response to this question.

Participants not completing either the IC or SCC (noncompleters) were asked to identify obstacles or issues they encountered that prevented course completion. Multiple-choice options offered included “Technical issues (e.g., computer, internet connection, website),” “Problems with navigating the modules,” “Lack of time or inability to schedule conveniently,” “Irrelevant/unimportant course content/topics,” “Low quality/attractiveness of course pages/videos/materials,” “Health related to MS,” “Other health issues,” “Family or work issues,” “Participation in another course or intervention,” “Did not receive reminder emails,” and “Other (specify in text).” Participants could select more than one response to this question.

### Participants’ Levels of Satisfaction With the MSOC

Completers were queried regarding their satisfaction with the corresponding courses. This included two questions related to (1) overall course experience, measured on a 6-point Likert scale from “excellent” to “very poor” (responses were categorized into “Excellent/very good,” “Good/average,” and “Poor/very poor”); and (2) how likely they would be to recommend the course to a peer or family member with MS, measured on a 5-point Likert scale ranging from “Extremely likely” to “Extremely unlikely” (responses were categorized into “Extremely/Somewhat likely,” “Neither,” and “Extremely/Somewhat unlikely”).

### Familiarity With the Course Content and Additional Information Seeking

Completers were asked to rate their familiarity with the course content before commencing the course using a 5-point Likert scale with options from “All of the content was new to me” to “I was familiar with all of the content.” Responses were categorized into “Familiar with most/all the content,” “Some of the content was new,” and “All/most of the content was new.”

Completers were also asked to select from a list of topics related to each module that they would like to receive additional information on (eg, “What is MS,” “Sunlight and vitamin D,” and “Exercise”; *yes* or *no*).

### Community Forum Engagement

We assessed engagement with the facilitator-run community forum by querying completers on whether they participated in the forum during the course (yes or no) and, if so, whether they found the community forum beneficial (yes or no), with text options provided to expand on these responses.

### Intention to Adopt Lifestyle Change Recommendations

To assess whether course engagement could facilitate lifestyle modification, completers were asked to rate their likelihood of adopting the lifestyle recommendations of the IC or SCC. The following question was asked for each topic or module: “As a result of taking the course, how likely are you to change the following lifestyle behaviour? (diet, smoking cessation, omega-3 supplementation, sun exposure or vitamin D supplementation, physical activity, meditation, and other stress reduction activities).” Responses were rated on a 5-point Likert scale: “extremely unlikely,” “moderately unlikely,” “neither,” “moderately likely,” and “extremely likely,” resulting in a total score ranging from 0 to 5. Participants selected “not applicable” if they already adhered to the lifestyle recommendation (eg, nonsmoking).

### Statistical Analysis

#### Participant Characteristics and Group Engagement

Demographic characteristics of the study participants at baseline were examined as sample sizes and percentages (categorical variables) and means and SDs (continuous variables). Motivators of and barriers to course completion, course satisfaction levels, engagement with the community forum, interest in seeking additional information on course topics, and intention to adopt lifestyle recommendations of the IC and SCC were examined as percentages. Differences between the IC and SCC groups were assessed using the 2-tailed *t* test for continuous variables and chi-square tests for categorical variables.

#### Assessing Factors of Intention to Implement Lifestyle Changes

We examined whether participant- or course-related factors were associated with participants’ intention to adopt individual lifestyle recommendations among course completers. Multivariate linear regression models were conducted to estimate adjusted regression coefficients and 95% CIs adjusting for baseline engagement with lifestyle recommendations and participation in another lifestyle or intervention program. To assess differences between the IC and SCC, we conducted moderation analyses by adding an interaction term (group × lifestyle behavior) to the regression models. All statistical analyses were conducted using Stata (version 17.0; StataCorp). Statistical significance was set at *P*<.05.

## Results

### Participant Characteristics

**Figure 1:**
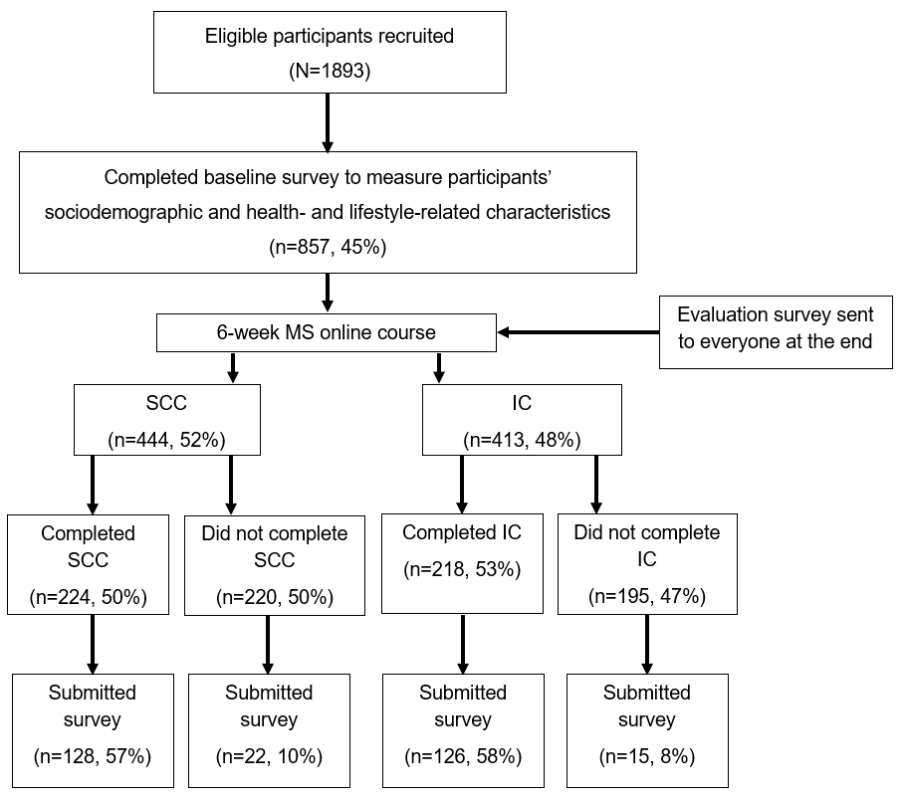
CONSORT (Consolidated Standards of Reporting Trials) flow diagram. IC: intervention course; MS: multiple sclerosis; SCC: standard-care course.

In total, 857 participants (aged ≥18 years with a confirmed clinical diagnosis of MS) enrolled in the MSOC effectiveness RCT and completed the baseline survey (IC: n=413, 48.2%; SCC: n=444, 51.8%; Figure 1). The analysis cohort comprised 291 participants (n=254, 87.3% course completers and n=37, 12.7% course noncompleters) who completed the baseline survey and postcourse evaluation survey.

There were similar numbers of participants (141/291, 48.5% IC participants and 150/291, 51.5% SCC participants) across both study arms (Table 1). Participants were 87.6% (255/291) women, and the mean age was approximately 50 years (SCC: mean 50.6, SD 10.7 years; IC: mean 49.8, SD 11.5 years). On the basis of visual inspection alone, participants’ sociodemographic characteristics appeared similar across both study arms except for BMI—the percentage of participants with obesity was lower in the IC (28/141, 19.9%) than in the SCC (48/150, 32%). A large proportion of participants held a university degree (187/291, 64.3%) and were partnered (210/291, 72.2%). Approximately half (141/291, 48.5%) of the participants were employed. The most frequent regions of residence were North America and Australia and New Zealand (Table S2 in Multimedia Appendix 4).

**Table 1:** Descriptive statistics of the study sample (N=291)^a^.

			SCC^b^ (n=150)	IC^c^ (n=141)
**Sociodemographic characteristics**				
	Gender (men), n (%)		15 (10.1)^d^	21 (14.9)
	Age (y), mean (SD)		50.6 (10.7)	49.8 (11.5)
	Educational level (lower than university), n (%)		58 (38.9)^d^	45 (31.9)
	University education, n (%)		91 (61.1)^d^	96 (68.1)
	Employment status (working), n (%)		67 (47.9)^e^	74 (56.9)^f^
	Partnered (married or de facto partnership), n (%)		109 (73.7)^g^	101 (71.6)
	**Country of residence, n (%)**			
		Australia or New Zealand	44 (29.5)^d^	41 (29.1)
		North America (United States or Canada)	55 (36.9)^d^	49 (34.8)
		United Kingdom	20 (13.4)^d^	20 (14.2)
		Other	30 (20.1)^d^	31 (22.0)
**Health characteristics**				
	MS^h^ type (nonprogressive), n (%)		105 (74.5)^i^	100 (76.9)^f^
	MS duration (y), mean (SD)		9.6 (8.43)	10.0 (8.87)
	Comorbidities (≥1), n (%)		83 (58.9)^i^	77 (57.9)^j^
	**BMI, n (%)**			
		Underweight or normal weight	65 (43.6)^d^	82 (58.6)^e^
		Overweight	36 (24.2)^d^	30 (21.4)^e^
		Obese	48 (32)^d^	28 (19.9)^k^
	Taking MS-related medication, n (%)		106 (71.6)^g^	88 (63.3)^l^
**Patient-reported outcome measures** **, n (%)**				
	**Disability (PDDS^m^)**			
		Normal or mild	72 (48.3)^d^	72 (51.1)
		Moderate	63 (42.3)^d^	52 (36.9)
		Severe	14 (9.4)^d^	17 (12.1)
	Clinically significant fatigue (mean FSS^n^>5)		80 (54.1)^g^	79 (56.8)^l^
	**Depression (HADS-D^o^)**			
		Normal	90 (62.1)^p^	93 (67.4)^q^
		Borderline	32 (22.1)^p^	24 (17.4)^q^
		Abnormal	23 (15.9)^p^	21 (15.2)^q^
	**Anxiety (HADS-A^r^)**			
		Normal	69 (47.3)^s^	68 (49.3)^q^
		Borderline	30 (20.6)^s^	40 (29)^q^
		Abnormal	47 (32.2)^s^	30 (21.7)^q^

^a^Percentages are based on available data; totals may be smaller due to missing values.

^b^SCC: standard-care course.

^c^IC: intervention course.

^d^n=149.

^e^n=140.

^f^n=130.

^g^n=148.

^h^MS: multiple sclerosis.

^i^n=141.

^j^n=133.

^k^*P*<.05.

^l^n=139.

^m^PDDS: Patient-Determined Disease Steps.

^n^FSS: Fatigue Severity Scale.

^o^HADS-D: Hospital Anxiety and Depression Scale–Depression subscale.

^p^n=145.

^q^n=138.

^r^HADS-A: Hospital Anxiety and Depression Scale–Anxiety subscale.

^s^n=146.

Most participants had nonprogressive MS types, and the mean time since diagnosis was approximately 10 years (SCC: mean 9.6, SD 8.43 years; IC: mean 10.0, SD 8.87 years). Approximately half of the participants reported no or mild disability (144/291, 49.5%) or clinically significant fatigue (159/291, 54.6%). On average, participants reported at least one comorbidity that was either physical or mental health–related. Approximately two-thirds of the participants reported symptoms of depression (183/291, 62.9%) and were taking medication to manage their MS (194/291, 66.7%), and 47.1% (137/291) had symptoms of anxiety within the normal range.

### Motivation for and Barriers to Completion

Among the 857 participants who completed the baseline survey, course completion rates were 50.5% (224/444) for the SCC and 52.8% (218/413) for the IC (Figure 1). Among the course completers, 57.1% (128/224) and 57.8% (126/218) of SCC and IC participants, respectively, completed the postcourse evaluation survey (Table 2). The main motivation for course completion was similar between both groups regarding the “opportunity to participate in MS research” (SCC: 104/128, 81.3%; IC: 96/126, 76.2%). Other commonly reported motivators of course completion included “topics were relevant” (SCC: 88/128, 68.8%; IC: 89/126, 70.6%), “convenience/flexible time” (SCC: 88/128, 68.8%; IC: 83/126, 65.9%), “to optimize my health” (SCC: 77/128, 60.2%; IC: 86/126, 68.3%), and “the course was interesting” (SCC: 67/128, 52.3%; IC: 68/126, 54%). Less common motivators of completion were “connection and interaction with others” (SCC: 7/128, 5.5%; IC: 20/126, 15.9%) and “engagement with facilitators” (SCC: 8/128, 6.3%; IC: 14/126, 11.1%).

Of the 417 participants who did not complete the course, 37 (8.9%) completed the evaluation survey (22/222, 9.9% SCC noncompleters and 15/195, 7.7% IC noncompleters; Figure 1). Among noncompleters, the most commonly reported barriers to course completion were “lack of time or inability to schedule conveniently” (SCC: 9/22, 41%; IC: 7/15, 47%) and “technological issues” (SCC: 7/22, 32%; IC: 2/15, 13%). Other barriers to completion were “health issues” and “family or work-related issues,” and low quality or attractiveness (ie, the course content lacked visual appeal or was not interesting) was reported by 14% (3/22) of SCC participants but no IC participants.

**Table 2:** Most common reasons for course completion and noncompletion^a^.

		SCC^b^, n (%)	IC^c^, n (%)
**Motivators among course completers**			
	Opportunity to participate in MS^d^ research	104 (81.3)^e^	96 (76.2)^f^
	Topics were relevant	88 (68.8)^e^	89 (70.6)^f^
	Convenient or flexible time	88 (68.8)^e^	83 (65.9)^f^
	To optimize their health	77 (60.2)^e^	86 (68.3)^f^
	The course was interesting	67 (52.3)^e^	68 (54)^f^
	Connection and interaction with others	7 (5.5)^e^	20 (15.9)^f,g^
	Engagement with facilitators	8 (6.3)^e^	14 (11.1)^f^
	Other	10 (7.8)^e^	10 (7.9)^f^
**Barriers to starting or completing the course among noncompleters**			
	Lack of time or inability to schedule time for the course conveniently	9 (40.9)^h^	7 (46.7)^i^
	Technological issues	7 (31.8)^h^	2 (13.3)^i^
	Health issues	3 (13.6)^h^	4 (26.7)^i^
	Family or work-related issues	1 (4.5)^h^	4 (26.7)^i^
	Low quality or attractiveness of the course	3 (13.6)^h^	0 (0)^i^
	Irrelevant or unimportant content or topics	2 (9.1)^h^	1 (6.7)^i^
	Problems navigating the modules	1 (4.5)^h^	1 (6.7)^i^
	Participation in another course or intervention	1 (4.5)^h^	1 (6.7)^i^
	Did not receive reminder emails	0 (0)^h^	1 (6.7)^i^
	Other	2 (9.1)^h^	0 (0)^i^

^a^Differences between the standard-care course and the intervention course were assessed using the chi-square test.

^b^SCC: standard-care course.

^c^IC: intervention course.

^d^MS: multiple sclerosis.

^e^n=128.

^f^n=126.

^g^*P*<.05.

^h^n=22.

^i^n=15.

### Participant Satisfaction

Most course completers were satisfied with the course across both study arms (78/128, 60.9% of SCC completers vs 92/126, 73% of IC completers rated the course as “excellent/very good,” and 48/128, 37.5% of SCC completers vs 32/126, 25.4% of IC completers rated it as “good/average”; Table 3). Less than 2% of completers across both study arms (SCC: 2/128, 1.6%; IC: 2/126, 1.6%) rated the course as “poor/very poor.” Most course completers (SCC: 106/128, 82.8%; IC: 113/126, 89.7%) were “extremely likely” or “somewhat likely” to recommend the course to friends or family members with MS, whereas approximately 4% (SCC: 5/128, 3.9%; IC: 5/126, 4%) were “extremely unlikely” or “somewhat unlikely” to recommend the course.

**Table 3:** Course completers’ evaluation of the intervention course (IC) and standard-care course (SCC) in terms of course satisfaction, course recommendation, and familiarity with the contenta.

Survey query		SCC (n=128), n (%)	IC (n=126), n (%)
**Overall satisfaction**			
	Excellent or very good	78 (60.9)	92 (73)
	Good or average	48 (37.5)	32 (25.4)
	Poor or very poor	2 (1.6)	2 (1.6)
**Recommendation to friend or family member with MS^b^**			
	Extremely or somewhat likely	106 (82.8)	113 (89.7)
	Neither	17 (13.3)	8 (6.3)
	Extremely or somewhat unlikely	5 (3.9)	5 (4)
**Familiarity with the content before MSOC^c^**			
	Familiar with most of or all the content	102 (79.7)	87 (69)^d^
	Some of the content was new	22 (17.2)	27 (21.4)^d^
	All or most of the content was new	4 (3.1)	12 (9.5)^d^

^a^Differences between the SCC and IC were assessed using the chi-square test.

^b^MS: multiple sclerosis.

^c^MSOC: Multiple Sclerosis Online Course.

^d^*P*<.05.

### Familiarity With Course Content and Information Seeking

More SCC completers (102/128, 79.7%) compared with IC completers (87/126, 69%) were familiar with some or all the course content before taking the course, and only 3.1% (4/128) of SCC completers and 9.5% (12/126) of IC completers found that all or most of the course content was new (Table 3). When course completers were queried as to whether they would like to receive additional information on each course topic, the most common topics selected by ≥24% of SCC and IC completers were “diet,” “physical activity (exercise),” “stress management,” “medication and family prevention,” and “review and consolidation” (Table 4). There was less interest in receiving additional information on “what is MS” (SCC: 12/128, 9.4%; IC: 16/125, 12.8%) and sun exposure (SCC: 24/128, 18.8%; IC: 17/125, 13.6%). Key topics of interest that SCC completers expressed greater interest in receiving additional information on than IC completers included physical activity (SCC: 49/128, 38.3%; IC: 30/125, 24%; *P*=.01) and “review and consolidation,” which was part of the conclusion module (SCC: 63/128, 49.2%; IC: 32/125, 25.6%; *P*<.001).

**Table 4:** Topics that course completers would have liked additional information on^a^.

Topic	SCC^b^ (n=128), n (%)	IC^c^ (n=125), n (%)
What is MS^d^	12 (9.4)	16 (12.8)
Diet	56 (43.8)	48 (38.4)
Sunlight and vitamin D	24 (18.8)	17 (13.6)
Physical activity	49 (38.3)	30 (24)^e^
Stress management	62 (48.4)	46 (36.8)
Medication and family prevention	41 (32)	32 (25.6)
Review and consolidation	63 (49.2)	32 (25.6)^f^

^a^Differences between the intervention course and the standard-care course groups were assessed using chi-square tests.

^b^SCC: standard-care course.

^c^IC: intervention course.

^d^MS: multiple sclerosis.

^e^*P*<.01.

^f^*P*<.001.

### Community Forum Engagement

A lower proportion of participants in the SCC (32/128, 25%) than in the IC (55/126, 43.7%) engaged with the community forum within the course. Furthermore, most completers who engaged with the forum found it useful (SCC: 23/32, 72%; IC: 44/55, 80%), with reasons including “appreciated the opportunity to gain insights from others’ experiences,” “highlighted the benefits of receiving answers to their questions,” “provided valuable resources from facilitators,” and “appreciated the ability to share their own perspective and experiences.” In contrast, the 2 main reasons why completers did not find the forum useful included “the limited ability to directly reply to and engage with other course members” and “the low number of participants in the forums.”

### Participants’ Intention to Make Lifestyle Modifications

A large proportion of IC and SCC completers intended to adopt lifestyle recommendations, particularly diet, sun exposure, physical activity, and stress management (Table 5). However, a smaller proportion of SCC versus IC completers were “extremely likely” or “moderately likely” to make changes in their diet based on the corresponding course content (74/128, 57.8% vs 89/125, 71.2%; *P*=.04). SCC completers were also less likely to increase their sun exposure (62/124, 50% vs 82/124, 66.1%; *P*=.01), supplement with omega-3 (60/126, 47.6% vs 84/125, 67.2%; *P*=.004), and practice meditation (66/126, 52.4% vs 85/124, 68.5%; *P*=.009). Of note, the SCC did not contain specific recommendations regarding sun exposure, omega-3 supplementation, or meditation practice (Table S1 in Multimedia Appendix 4).

**Table 5:** Intentions of participants in the intervention course (IC) and the standard-care course (SCC) to implement lifestyle changes.

Lifestyle change	SCC (n=128)^a^, n (%)				IC (n=126)^b^, n (%)				*P* value
	Extremely likely or moderately likely	Neither, extremely unlikely, or moderately unlikely	Not applicable	Extremely likely or moderately likely		Neither, extremely unlikely, or moderately unlikely	Not applicable		
Diet	74 (58.3)^c^	45 (35.4)^c^	8 (6.3)^c^	89 (71.2)^d^		26 (20.8)^d^	10 (8.0)^d^	.04	
Vitamin D supplementation	49 (38.6)^c^	31 (24.4)^c^	47 (37.0)^c^	64 (52.5)^e^		20 (16.4)^e^	38 (31.2)^e^	.07	
Sun exposure	62 (50.0)^f^	45 (36.3)^f^	17 (13.7)^f^	82 (66.1)^f^		25 (20.2)^f^	17 (13.7)^f^	.01	
Omega-3 supplementation	60 (47.6)^g^	43 (34.1)^g^	23 (18.3)^g^	84 (67.2)^d^		22 (17.6)^d^	19 (15.2)^d^	.004	
Physical activity	84 (67.2)^d^	25 (20.0)^d^	16 (12.8)^d^	85 (69.1)^h^		20 (16.3)^h^	18 (14.6)^h^	.72	
Meditation	66 (52.4)^g^	46 (36.5)^g^	14 (11.1)^g^	85 (68.6) ^f^		24 (19.4) ^f^	15 (12.1) ^f^	.009	
Other stress reduction activities ^i^	84 (67.2)^d^	30 (24.0)^d^	11 (8.8)^d^	92 (75.4)^e^		23 (18.9) ^e^	7 (5.7)^e^	.34	
Nonsmoking	12 (9.5)^g^	14 (11.1)^g^	100 (79.4) ^g^	14 (11.5)^e^		8 (6.6)^e^	100 (82.0)^e^	.42	

^a^Percentages are based on available data only; subgroup totals may be smaller than 128 due to missing data.

^b^Percentages are based on available data only; subgroup totals may be smaller than 126 due to missing data.

^c^n=127.

^d^n=125.

^e^n=122.

^f^n=124.

^g^n=126.

^h^n=123.

^i^Stress reduction activities other than meditation.

### Factors Associated With Participants’ Intentions for Lifestyle Modifications

Multivariate analysis found associations between several course-related factors and completers’ intentions to adopt the lifestyle recommendations of each course (Table 6).

In particular, course satisfaction was associated with an intention to adopt vitamin D supplementation among SCC completers, whereas among IC completers, course satisfaction was associated with an intention to adopt dietary recommendations, vitamin D supplementation or sun exposure, and meditation practice. For example, IC completers who rated the course as “very good” or “excellent” had a 1.69-point (95% CI 0.24-3.13) higher intention to adopt a healthy diet due to the course. Engagement with the community forum, new course content, and seeking additional information on certain topics were also associated with intentions to change certain factors, with no group differences between the SCC and IC.

**Table 6:** Course-related, sociodemographic, and health-related factors associated with intentions to change certain lifestyle behaviors in standard-care course (SCC) and intervention course (IC) completers^a^.

Factor and course				aβ^b^ (95% CI)										
				Healthy diet		Vitamin D supplementation		Sun exposure		Omega-3 supplementation		Physical activity		Meditation
**Course related**														
	**High satisfaction^c^**													
		SCC	0.02 (−2.10 to 2.14)		0.86 (0.21 to 1.50)^d^		−0.31 (−2.41 to 1.80)		−*0.05 (−2.48 to 2.38)*^e^		0.18 (−1.95 to 2.32)		−0.45 (−2.84 to 1.94)	
		IC	1.69 (0.24 to 3.13)^d^		0.99 (0.49 to 1.50)^f^		3.39 (1.41 to 5.36)^g^		−*0.21 (−1.64 to 1.21)*		1.81 (−0.01 to 3.64)		1.54 (0.01 to 3.07)^d^	
	**Participated in forum**													
		SCC	0.98 (0.26 to 1.69)^g^		0.80 (−0.55 to 2.15)		0.56 (−0.49 to 1.61)		*0.67 (−0.47* *to* *1.80)*		1.20 (0.42 to 1.99)^g^		0.77 (−0.14 to 1.67)	
		IC	0.28 (−0.48 to 1.04)		−0.20 (−1.31 to 0.91)		1.02 (0.16 to 1.88)^d^		*0.11 (−0.76* *to* *0.98)*		0.67 (−0.26 to 1.60)		0.87 (0.14 to 1.60)^d^	
	**Learning new content^h^**													
		SCC	0.15 (−0.35 to 0.64)		0.06 (−0.72 to 0.84)		0.26 (−0.15 to 0.66)		*0.09 (−0.49* *to* *0.68)*		0.63 (0.06 to 1.19)^d^		0.58 (0.20 to 0.97)^g^	
		IC	0.56 (0.16 to 0.97)^g^		0.23 (−0.26 to 0.73)		0.52 (0.06 to 0.98)^d^		*0.42 (−0.00* *to* *0.84)*		0.33 (−0.31 to 0.97)		0.46 (0.04 to 0.88)^d^	
	**Seeking information**													
		SCC	0.55 (0.16 to 0.94)^g^		0.94 (0.24 to 1.65)^d^		0.22 (−0.19 to 0.64)		*0.50 (0.03* *to* *0.96)* ^d^		0.45 (0.06 to 0.85)^d^		0.00 (−0.48 to 0.49)	
		IC	0.55 (0.17 to 0.93)^g^		−0.05 (−0.75 to 0.65)		0.44 (−0.01 to 0.89)		*0.22 (−0.24* *to* *0.69)*		0.52 (0.10 to 0.95)^d^		0.42 (−0.07 to 0.90)	
**Sociodemographic**														
	**Age of >54 y**													
		SCC	−0.02 (−0.72 to 0.68)		0.43 (−0.77 to 1.63)		−0.30 (−0.99 to 0.40)		−*0.03 (−0.80 to 0.11)*		−0.85 (−1.51 to −0.16)^d^		−0.51 (−1.28 to 0.27)	
		IC	−0.52 (−1.26 to 0.21)		−0.38 (−1.25 to 0.49)		0.39 (−1.08 to 0.31)		*0.87 (0.10* *to* *1.64)* ^d^		−0.38 (−1.08 to 0.31)		−0.05 (−0.83 to 0.72)	
	**Working**													
		SCC	−0.20 (−0.62 to 0.22)		−0.25 (−0.91 to 0.41)		0.29 (−0.15 to 0.73)		−0.09 (−0.60 to 0.42)		0.02 (−0.40 to 0.45)		−*0.53 (−0.98* *to* *−0.07)*^d^	
		IC	0.20 (−0.22 to 0.62)		0.25 (−0.26 to 0.76)		0.13 (−0.31 to 0.57)		0.06 (−0.38 to 0.50)		0.39 (−0.03 to 0.81)		*0.45 (0.01* *to* *0.90)* ^d^	
	**University education**													
		SCC	*0.23 (−0.18* *to* *0.63)*		−0.45 (−1.10 to 0.19)		−0.01 (−0.43 to 0.41)		−*0.09 (−0.59* *to* *0.41)*		−0.16 (−0.57 to 0.26)		0.23 (−0.24 to 0.70)	
		IC	−*0.50 (−0.90* *to* *−0.07)*^d^		−0.35 (−0.87 to 0.17)		0.08 (−0.40 to 0.55)		−*0.58 (−1.02* *to* *−0.15)*^g^		−0.34 (−0.77 to 0.10)		−0.35 (−0.82 to 0.12)	
**Health related**														
	**Long MS^i^ duration**													
		SCC	0.65 (0.05 to 1.26)^d^		*0.97 (0.03* *to* *1.91)* ^d^		−0.27 (−0.90 to 0.35)		−0.15 (−0.91 to 0.61)		−0.19 (−0.83 to 0.46)		−0.06 (−0.74 to 0.63)	
		IC	−0.06 (−0.60 to 0.49)		*0.12 (−0.54* *to* *0.79)*		0.13 (−0.45 to 0.72)		0.03 (−0.51 to 0.57)		−0.12 (−0.64 to 0.40)		−0.40 (−0.97 to 0.17)	
	**Used DMTs^j^**													
		SCC	0.10 (−0.33 to 0.53)		0.04 (−0.64 to 0.72)		0.40 (−0.05 to 0.84)		0.26 (−0.27 to 0.78)		−0.18 (−0.63 to 0.27)		−0.44 (−0.93 to 0.04)	
		IC	0.44 (0.03 to 0.85)^d^		0.12 (−0.39 to 0.63)		0.03 (−0.41 to 0.46)		0.21 (−0.22 to 0.64)		0.26 (−0.14 to 0.67)		−0.19 (−0.64 to 0.26)	
	≥**1 comorbidity**													
		SCC	0.03 (−0.38 to 0.43)		0.10 (−0.67 to 0.67)		−0.21 (−0.65 to 0.23)		−0.14 (−0.66 to 0.36)		0.10 (−0.42 to 0.44)		−0.05 (−0.53 to 0.44)	
		IC	0.31 (−0.09 to 0.71)		0.06 (−0.45 to 0.58)		0.01 (−0.43 to 0.44)		0.50 (0.10 to 0.89)^d^		0.13 (−0.27 to 0.53)		0.33 (−0.13 to 0.78)	

^a^We assessed associations between sociodemographic and course- and health-related factors and participants’ intention to making changes in the healthy lifestyle behavior (range from 0=extremely unlikely to 4=extremely likely). Multiple linear regression models were used to estimate adjusted regression coefficients and 95% CIs adjusting for baseline lifestyle behaviors and involvement in other lifestyle interventions or programs. Differences between the SCC and IC were assessed using interaction analyses.

^b^aβ: adjusted regression coefficient.

^c^High satisfaction: participants who rated their overall satisfaction with the course as “very good” or “excellent.”

^d^*P*<.05 between factors and intentions for lifestyle change within the SCC or IC.

^e^ Group difference (*P*<.05) from SCC.

^f^*P*<.001 between factors and intentions for lifestyle change within the SCC or IC.

^g^*P*<.01 between factors and intentions for lifestyle change within the SCC or IC.

^h^Learning new content: participants who rated the course content as “most,” “all,” or “some” being new.

^i^MS: multiple sclerosis.

^j^DMT: disease-modifying therapy.

Sociodemographic factors associated with intentions for lifestyle modifications included age (being aged >54 years was associated with intentions to increase physical activity and supplement with omega-3, with the latter being significantly lower in SCC vs IC completers), employment status (being employed was associated with a higher intention to practice meditation among SCC completers and a lower intention among IC completers), educational level (a university degree was associated with a greater intention for diet changes and omega-3 supplementation among SCC vs IC completers).

Regarding health-related factors, longer MS duration (>15 years since diagnosis) was associated with a greater intention for dietary modifications and vitamin D supplementation among SCC versus IC completers. The use of DMTs and the presence of comorbidities were associated with intentions to adopt certain lifestyle modifications, with no significant differences between study arms.

Poorer participant-reported health outcomes at baseline were linked to higher intentions to adopt various lifestyle modifications, particularly among SCC completers (Table 7). Severe anxiety, depression and disability, and clinically significant fatigue were associated with greater intentions to adopt dietary modifications, vitamin D supplementation, increased sun exposure, and physical activity among SCC versus IC completers. However, higher physical and mental QoL was associated with lower intentions to modify vitamin D intake, omega-3 supplementation, and physical activity among SCC completers.

**Table 7:** Participant-reported health outcomes associated with intentions to change certain lifestyle behaviors among standard-care course (SCC) and intervention course (IC) completers^a^.

Outcome and course				aβ^b^ (95% CI)										
				Healthy diet		Vitamin D supplementation		Sun exposure		Omega-3 supplementation		Physical activity		Meditation
**Health outcomes**														
	**Severe anxiety^c^**													
		SCC	0.26 (−0.19 to 0.72)		0.51 (−0.20 to 1.23)		0.45 (−0.12 to 1.02)		0.65 (0.09 to 1.20)^d^		0.50 (−0.03 to 1.02)		*0.59 (0.08* *to* *1.10)* ^d,e^	
		IC	0.45 (−0.00 to 0.90)		−0.16 (−0.77 to 0.45)		0.19 (−0.29 to 0.67)		0.11 (−0.43 to 0.66)		0.08 (−0.37 to 0.52)		*0.06 (−0.50* *to* *0.62)*	
	**Severe depression^c^**													
		SCC	0.79 (0.25 to 1.34)^f^		0.97 (0.10 to 1.85)^d^		0.10 (−0.43 to 0.63)		0.33 (−0.26 to 0.92)		0.51 (0.01 to 1.01)^d^		0.12 (−0.45 to 0.68)	
		IC	0.42 (−0.15 to 1.00)		0.21 (−0.49 to 0.90)		0.13 (−0.39 to 0.66)		−0.04 (−0.56 to 0.47)		0.19 (−0.31 to 0.68)		0.06 (−0.61 to 0.72)	
	**Fatigue^g^**													
		SCC	0.05 (−0.35 to 0.46)		0.62 (0.03 to 1.24)^d^		0.38 (−0.04 to 0.79)		0.19 (−0.29 to 0.67)		0.20 (−0.21 to 0.61)		0.14 (−0.31 to 0.60)	
		IC	−0.01 (−0.41 to 0.40)		0.08 (−0.40 to 0.57)		0.02 (−0.40 to 0.43)		−0.07 (−0.48 to 0.34)		−0.18 (−0.58 to 0.22)		0.03 (−0.41 to 0.48)	
	**Severe disability^h^**													
		SCC	*0.80 (0.10 to 1.50)^d^*		*1.51 (0.43 to 2.59)* ^f^		0.48 (0.03 to 0.92)^d^		0.21 (−0.63 to 1.05)		*0.49 (0.06 to 0.91)* ^d^		0.78 (−0.06 to 1.62)	
		IC	−*0.42 (−0.83* *to* *−0.00)*^d^		−*0.51 (−1.47* *to* *0.35)*^d^		−0.14 (−0.58 to 0.30)		−0.03 (−0.70 to 0.64)		−*0.18 (−0.60 to 0.23)*		−0.31 (−1.15 to 0.54)	
	**Physical HRQoL^i^**													
		SCC	−0.00 (−0.02 to 0.01)		−*0.03 (−0.04 to −0.01)*^f^		0.01 (−0.00 to 0.02)		−0.01 (−0.02 to 0.00)		−0.01 (−0.02 to −0.00)^d^		−0.01 (−0.02 to 0.00)	
		IC	−0.01 (−0.01 to 0.01)		*0.01 (−0.01 to 0.02)*		−0.01 (−0.02 to 0.00)		0.00 (−0.01 to 0.01)		−0.00 (−0.01 to 0.01)		−0.00 (−0.02 to 0.01)	
	**Mental HRQoL^i^**													
		SCC	−0.00 (−0.01 to 0.01)		−*0.02 (−0.04 to −0.01)*^f^		−0.01 (−0.02 to 0.00)		−0.01 (−0.02 to −0.00)^d^		−0.01 (−0.02 to −0.00)^d^		−0.01 (−0.02 to 0.00)	
		IC	−0.01 (−0.02 to 0.00)		*0.01 (−0.01 to 0.01)*		0.00 (−0.01 to 0.01)		−0.01 (−0.02 to 0.00)		−0.00 (−0.01 to 0.01)		0.00 (−0.01 to 0.01)	

^a^We assessed associations between health outcomes and participants’ intention of making changes to the healthy lifestyle behavior (range from 0=extremely unlikely to 4=extremely likely) in each course. Multiple linear regression models were used to estimate adjusted regression coefficients and 95% CIs adjusting for baseline lifestyle behaviors and involvement in other lifestyle interventions or programs. Differences between the SCC and IC were assessed using interaction analyses.

^b^aβ: adjusted regression coefficient.

^c^Hospital Anxiety and Depression Scale.

^d^*P*<.05 between health outcomes and intentions for lifestyle change within the SCC or IC.

^e^ Group difference (*P*<.05) from SCC.

^f^*P*<.01 between health outcomes and intentions for lifestyle change within the SCC or IC.

^g^Clinically significant fatigue (Fatigue Severity Scale).

^h^Patient-Determined Disease Steps.

^i^HRQoL: health-related quality of life.

## Discussion

### Principal Findings

Despite the potential advantages of digital health interventions for people living with MS in terms of accessibility and cost-effectiveness, their engagement with web-based interventions is low [18]. This study comprehensively evaluated factors associated with engagement with a novel web-based lifestyle-related program, the MSOC, by people living with MS and examined short-term outcomes following course completion nested within the flagship RCT. Findings revealed that participants in both study groups (SCC and IC) found the course content interesting and useful and reported high levels of course satisfaction. Importantly, there was evidence that the course promoted intentions to implement lifestyle behavior changes, and key factors associated with these intentions were identified across both study arms. These findings provide new insights for designing and implementing web-based lifestyle interventions tailored to the MS community, addressing critical gaps in usability, engagement, and learnability of digital health solutions.

### Course Completion

We observed completion rates of 23.3% (442/1893) for enrolled participants and 51.6% (442/857) among those who completed the baseline survey. These completion rates are noteworthy considering that participants were required to complete a 166-question baseline survey, and a previous scoping review reported 5% to 15% completion rates of health education massive open online courses (MOOCs) by the general population [35]. Given the documented desire of people living with MS to receive lifestyle-related information to improve their future health prospects [25], this may explain the higher rates of completion in this study. Similarly, this cohort enrolled in the RCT may represent a subgroup of highly motivated people living with MS.

### Motivators of and Barriers to Completion

Primary factors contributing to course completion included the “opportunity to participate in MS research” and “to optimize my health.” These findings align with those of the MSOC feasibility study [26], where many people living with MS reported that these factors were crucial to their engagement with the course. The intention of people living with MS to contribute to MS research is consistent with previous findings and represents one of the most common reasons why individuals engage in any research [27]. In addition, participants considered relevant course content and the flexibility to engage with the course in their own time as factors that influence course completion.

Primary barriers to completion were time constraints and technical issues. Other completion barriers included health-, family-, and work-related commitments. While MS-related studies examining noncompletion of web-based courses are limited, a recent mixed methods study of a 6-week MOOC called Understanding MS aimed at increasing understanding and awareness of MS also found that time constraints and MS-related symptoms were common completion barriers [36]. Other studies have also identified barriers to the uptake of web-based health interventions, such as older age, lower educational level, and MS symptoms [37].

### Course Satisfaction, Familiarity, and Seeking of Additional Information

While course satisfaction was high across both study arms, satisfaction levels were higher in the IC arm than in the SCC arm (IC: 92/126, 73%; SCC: 78/128, 60.9%). Due to the quantitative nature of our evaluative study, we can only speculate why participants found the course satisfactory. Plausible reasons for high course satisfaction may be participants’ strong interest in obtaining information on healthy lifestyle recommendations or self-management strategies to improve their future health prospects, as found in the qualitative studies of this trial, where participants expressed that they sought new knowledge to empower them to self-manage their MS [21,22].

The positive tone of the MSOC may have further contributed to the high satisfaction rates as this was found to be an important factor in the qualitative aspect of the MSOC feasibility RCT [38,39]. Similarly, “keep it positive” was 1 of the 3 core principles for developing the 6-week Understanding MS MOOC [40].

Participants in the SCC arm were more familiar with the course content and expressed greater interest in receiving additional lifestyle-related information than those in the IC. This is not surprising due to the SCC course content being sourced from publicly available MS society websites, whereas the content of the IC was novel for many IC participants. People living with chronic illnesses such as MS often exhibit a strong desire for new information to enhance their understanding and management of their condition [41]. Seeking additional information may reflect participants’ motivation and engagement with digital health interventions aimed at disease self-management. Similarly, digital interventions for diabetes and rheumatoid arthritis have shown that reliable, professional guidance is particularly valued, especially by newly diagnosed individuals [42,43].

Interestingly, while the SCC provided information from readily available sources such as international MS websites, only a small proportion of participants (SCC: 2/128, 1.6%; IC: 2/126, 1.6%) were dissatisfied with the course, and a substantial proportion (SCC: 106/128, 82.8%; IC: 113/126, 89.7%) indicated that they would recommend the course to others. These findings are consistent with those of the MSOC feasibility study [39], where the MSOC was found to meet participants’ expectations regardless of their familiarity with the course materials.

While new information that addresses knowledge gaps is critical for engaging participants in digital interventions, current findings suggest that even those familiar with the material can benefit when interventions provide fresh perspectives and dynamic delivery formats. Digital health interventions that offer updated, evidence-based content tailored to diverse participant needs—ranging from foundational knowledge for beginners to advanced updates for experienced users—are likely to achieve higher satisfaction and engagement.

### Community Forum Engagement

In the previous MSOC feasibility RCT, there was no engagement with the forum [39]. Although participants expressed positivity regarding the advantages of the forum in terms of connecting with other people with MS, they felt nervous and were reluctant to initiate discussions. This limitation was anticipated to potentially affect course completion as a pilot RCT of a web-based self-management program for fatigue for people living with MS found that lower interaction and support were associated with increased study dropout [12]. To address this issue in the current MSOC effectiveness RCT, a researcher facilitator was introduced into the community forum in each course. The facilitator, who was familiar with the evidence-based recommendations and course content, was responsible for initiating conversations, creating discussions, and answering participants’ queries to encourage participant interaction and, ultimately, course completion. Subsequently, we found that 25% (32/128) and 43.7% (55/126) of participants in the SCC and IC study arms, respectively, engaged with the forum in this study.

Notably, the engagement with the facilitator-led community forum did not significantly improve course completion rates (IC: 218/413, 52.8%; SCC: 224/444, 50.5%) compared to the feasibility study (59% in the IC and 50% in the SCC). Furthermore, facilitators were cited as a motivating factor for course completion by only a small proportion of participants (SCC: 8/128, 6.3%; IC: 14/126, 11.1%), although limited forum engagement, with less than half of the participants actively taking part, likely constrained its impact on course completion. Key barriers to forum engagement identified by the participants included the inability to directly reply to one another (restricted to only replying in a group thread) and the small number of forum participants. As shown in previous digital health interventions, participants with neurological disorders particularly value emotional support from others with similar conditions [44]. Addressing these restrictions could create more opportunities for meaningful interaction and enhance the potential benefits of peer support in future iterations.

Interestingly, among those who did participate in the forum, most found it helpful (SCC: 23/32, 72%; IC: 44/55, 80%), particularly for accessing shared resources and gaining insights from others. These findings align with the findings of the 2 qualitative studies related to this RCT [21,22] and existing evidence [45] suggesting that participants are more likely to engage with digital health interventions that promote both social connectedness and information sharing.

Overall, while facilitator-led forums hold promise, the presence of a facilitator alone may not be sufficient to drive higher course completion rates. Introducing structured activities or prompts could further encourage engagement and foster dynamic conversations that extend beyond a small subset of participants.

### Participants’ Intentions to Undertake Lifestyle Modifications

The overarching aim of the MSOC was to improve QoL and health outcomes in people living with MS. Preliminary evaluation found that course completers across both study arms intended to initiate lifestyle modifications following the course. According to the theory of planned behavior [46], intentions serve as crucial predictors for behavior change, thereby providing support for the potential of the MSOC to encourage lifestyle modification in people living with MS. This will be assessed in detail by analyzing primary and secondary outcomes of the RCT at the 6-, 12-, 24-, and 30-month follow-ups.

Across both study arms, physical activity and stress reduction activities that did not include meditation practice were the most common lifestyle recommendations that participants intended to adopt. Notably, significantly more IC completers intended to increase their sun exposure, supplement with omega-3, and practice meditation than SCC completers. This finding was expected considering that no specific recommendations regarding these lifestyle modifications were provided in the SCC. In contrast, the SCC provided more general dietary information, emphasizing the importance of a balanced diet and providing information on the vast range of MS-related diets adopted by people living with MS.

A recent systematic review of web-based health education interventions found that most included studies focused on knowledge gain as the main outcome, whereas the impact of knowledge gain on other outcomes such as lifestyle modification was less clear [18]. However, the Understanding MS MOOC study did examine lifestyle modification in people living with MS 8 to 10 weeks after course completion and found that 43% to 52% of participants had made improvements in their diet quality, increased their physical activity, and initiated supplementation with vitamin D [47]. Of note, these modifications were observed despite only 1 of the 6 MOOC learning modules providing information on lifestyle-related risk factors. Collectively, the aforementioned study combined with our study findings demonstrates that web-based learning can potentially facilitate lifestyle modification in people living with MS. The findings are consistent with meta-analytic evidence of the effects of web-based lifestyle interventions in improving diet quality and increasing physical activity in survivors of cancer [48] and people with cardiovascular disease [49].

### Factors Associated With Intentions to Undertake Lifestyle Modifications

Understanding factors that may influence individuals’ experiences with web-based lifestyle interventions is important in optimizing the effectiveness of tailored programs. In this study, course-related factors were associated with participants’ immediate learning outcomes. For instance, higher course satisfaction and an interest in seeking new information correlated with a greater intention to implement lifestyle modifications across both study arms, consistent with previous reports of links between knowledge gain and increased motivation [50].

Engagement with the forum was also associated with participants’ intentions to change certain lifestyle behaviors, consistent with previous study findings indicating that social connections made with peers during educational programs are important for engagement [51,52] and can mediate lifestyle changes [53]. In particular, a recent systematic review found that human support was a crucial component in the effectiveness of web-based behavior change interventions [19]. Furthermore, engagement with health care professionals in an MS-related face-to-face lifestyle program was found to increase participants’ sense of control and agency in adopting lifestyle changes [52,54]. Overall, these results highlight the importance of facilitator-led community forums as integral components of digital health interventions. With tailored design and implementation, these features could be optimized to promote increased health-related knowledge and support lifestyle changes.

Of interest, a large proportion of SCC completers expressed their intention to change their lifestyle despite being familiar with the course content. Therefore, the SCC may have served to refresh or reinforce their existing knowledge. Engagement with information presented in a different setting or format (eg, animation and videos) may also have been a motivating factor for facilitating intentions for lifestyle changes. Alongside the observed high satisfaction levels, these results highlight the perceived value of the MSOC irrespective of participants’ previous familiarity with the course materials, translating acquired or refreshed knowledge into motivational changes. This insight offers a key recommendation for future digital health design: developing modules that cater to both beginners and those with previous knowledge. For participants already familiar with the content, modules can serve as a refresher or provide new perspectives through web-based and visually engaging formats such as animations, videos, or quizzes.

Interestingly, other participant-related factors were also associated with intentions for lifestyle change. For instance, higher educational level was associated with lower intention for lifestyle change, consistent with studies indicating that participants with lower educational levels find course content more informative and applicable [17,55]. This highlights the importance of using clear, practical messaging and simplifying language and concepts to ensure accessibility for a broad audience. Disability was associated with an intention for diet change, vitamin D supplementation, and increased physical activity in the SCC but not in the IC. This may be attributed to the content of the SCC as it may not be possible for someone with a severe disability to adopt the restrictive diet or physical activity recommendations of the IC, especially if they rely on carers. As a result, it is important to consider course content and its applicability to people living with MS of all abilities.

In addition, SCC participants with severe mental health issues and lower QoL reported higher intentions to adopt lifestyle modifications. However, interestingly, our analysis of baseline characteristics and course completion in this RCT found that those with poor health status at baseline were less likely to complete the course [20]. While participants with poorer health may face physical or psychological challenges that reduce their likelihood of completing web-based courses, collectively, our results suggest that participants who complete web-based courses have a higher likelihood of implementing behavior change. Moreover, these findings suggest that future digital health interventions need to be designed to attempt to overcome course noncompletion, possibly by implementing tailored and motivational strategies such as progress tracking, personalized feedback, and interactive support tools to help overcome barriers to engagement to maximize the benefits of these programs.

### Strengths and Limitations

This study sample represents a large and diverse international cohort of people living with MS, with characteristics similar to those of other international cohorts [7,37]. The findings expand on previously documented qualitative insights from this trial [21,22], providing a more comprehensive understanding of user experiences and engagement with web-based educational programs. The MSOC’s utility as a web-based program demonstrates the potential of digital interventions to reach large, geographically diverse populations. This scalability is particularly beneficial for other chronic conditions for which health care access may be limited.

Various limitations should be considered. First, the study sample was restricted to RCT participants who completed the baseline and evaluation surveys. As research on web-based health interventions for chronic illnesses is likely to face challenges in recruiting and retaining participants [16,55], our study sample may be biased toward healthier individuals or those highly motivated to acquire lifestyle-related knowledge and adopt lifestyle changes. Second, while efforts were undertaken to recruit participants through various channels worldwide, this study also primarily involved participants from English-speaking countries with convenient internet able to access the recruitment sites. Consequently, the generalizability of the results may be limited, warranting caution in their interpretation. Third, the sample size of noncompleters across study arms may be considered small. This increases the risk of selection bias, potentially limiting the generalizability of the findings to a broader population of noncompleters. Nevertheless, identifying barriers to course completion, such as time limitations and technical and health-related issues, provides important insights for informing the development of web-based programs. For example, extending the course to >6 weeks may allow more participants to complete it, a strategy supported by findings from the Understanding MS MOOC study [36]. Other considerations may include presenting participants with incentives to complete the MSOC, such as completion certificates [56]. Furthermore, it is important to consider health disparities within the MS community to ensure that course content is appropriate for web-based interventions.

### Conclusions

The evaluation of the MSOC in this study provided new and valuable insights for the development of future web-based programs for people living with MS. Participants expressed high levels of satisfaction with the MSOC and strong intentions to adopt lifestyle modifications, even among those already familiar with the course content. Notably, course completers with poorer health had stronger intentions to adopt recommended lifestyle changes upon completing the course, underscoring the potential value of web-based interventions among those most likely to benefit. Identifying motivators and barriers to web-based course engagement could inform future course development to facilitate increased engagement and potentially achieve intervention objectives. Subsequent follow-up studies of the RCT’s primary and secondary outcomes will examine the effectiveness of the MSOC intervention in supporting the adoption and maintenance of lifestyle changes and achieving the primary outcome of the study (clinically significant improvements in QoL). Overall, tailored, accessible, and innovative web-based interventions offer an important platform to assist people living with MS in self-managing their MS and related symptoms and potentially improving their future health prospects.

## Appendix

Multimedia Appendix 1 Postcourse evaluation questions for participants.

Multimedia Appendix 2 Microsoft PowerPoint (Microsoft Corp) presentation of the conference talk about the Multiple Sclerosis Online Course.

Multimedia Appendix 3 Screenshots of the Multiple Sclerosis Online Course website.

Multimedia Appendix 4 Content of the standard-care course and intervention course and countries of residence of Multiple Sclerosis Online Course participants.

Multimedia Appendix 5 CONSORT-eHEALTH (V 1.6.1) checklist.

## Abbreviations

DMT: disease-modifying therapy
IC: intervention course
MOOC: massive open online course
MS: multiple sclerosis
MSOC: Multiple Sclerosis Online Course
MSQOL-54: Multiple Sclerosis Quality of Life–54
QoL: quality of life
RCT: randomized controlled trial
SCC: standard-care course

## Data Availability

The datasets generated or analyzed during this study are available from the corresponding author on reasonable request.
