# Supplementary material for: A Web-Based Lifestyle-Related Course for People Living With Multiple Sclerosis: Quantitative Evaluation of Course Completion, Satisfaction, and Lifestyle Changes Among Participants Enrolled in a Randomized Controlled Trial
Source: JMIR Hum Factors. 2025 May 26;12:e59363. doi: 10.2196/59363 (PMC12149781; doi:10.2196/59363)
Supplement: Multimedia Appendix 1 [file humanfactors_v12i1e59363_app1.pdf]

MSOC evaluation survey

The team would like to thank you for your interest and support in the Multiple Sclerosis Online Course study! Your feedback will help us to evaluate the effectiveness of the course, make improvements, and provide valuable resources for people living with MS.

Please enter the same name and email address you used in the course, this will help researchers to match your data for analysis. Thank you!

Q What is your full name?

Q What is your email address?

Q Did you **start** the course?

- ◯ Yes
- ◯ No

Q Did you **complete** the course?

- ◯ Yes
- ◯ No
- ◯ Can't remember

Q What **barriers or issues** hindered you from **starting** the course (you may select more than one answer)?

- ☐ Technical issues (e.g., computer, internet connection, website)
- ☐ Difficulty with the enrolment process
- ☐ Having to complete the baseline survey
- ☐ Lack of time or inability to schedule conveniently
- ☐ Health issues related to your MS
- ☐ Other health issues
- ☐ family or work commitments
- ☐ Participation in another course or intervention
- ☐ Other (please specify)

____

Q If you had any difficulty with the course or enrolment process, what would have made it easier for you?

____

Q What **barriers or issues** hindered you from **completing** the course (you may select more than one answer)?

- ☐ Technical issues (e.g., computer, internet connection, website)
- ☐ Problems with navigating the modules
- ☐ Lack of time or inability to schedule conveniently
- ☐ Irrelevant/unimportant course content/topics
- ☐ Low quality/attractiveness of course pages/videos/materials
- ☐ Health related to your MS
- ☐ Other health issues
- ☐ Family or work issues
- ☐ Participation in another course or intervention
- ☐ Did not receive reminder emails
- ☐ Other (please specify)

____

Q What **motivated** you to **complete** the course (you may select more than one answer)?

- [ ] Convenient/flexible time and location
- [ ] Opportunity to participate in MS research
- [ ] Topics that were relevant and/or important to me
- [ ] The course was interesting and/or easy to navigate
- [ ] Connection and interaction with other people with MS
- [ ] Engagement with facilitators
- [ ] To optimise my health
- [ ] Other (please specify)

____

Q What **motivated** you to **take** the course (you may select more than one answer)?

- [ ] Convenient/flexible time and location
- [ ] Opportunity to participate in MS research
- [ ] Topics that were relevant and/or important to me
- [ ] The course was interesting and/or easy to navigate
- [ ] Connection and interaction with other people with MS
- [ ] Engagement with facilitators
- [ ] To optimise my health
- [ ] Other (please specify)

____

Q How did you **first hear** of the Multiple Sclerosis Online Course (MSOC) study?

- ○ MS society websites (1)
- ○ Clinical trial website
- ○ MS society or MS organisation websites
- ○ Facebook advertisements
- ○ Twitter or Instagram advertisements
- ○ MS forums or other online social groups (please specify)
- ○ mouth from friend or family
- ○ Another way, please specify

____

- ○ Don't remember

Q Rate your **overall experience** of the course?

- ○ Excellent
- ○ Very good
- ○ Good
- ○ Average
- ○ Poor
- ○ Very poor
- ○ Unable to assess as did not complete sufficient amount of course

Q Rate your **overall experience** of the course?

- ◯ Excellent
- ◯ Very good
- ◯ Good
- ◯ Average
- ◯ Poor
- ◯ Very poor

Q How likely are you to **recommend** this course to a friend/family with MS?

- ◯ Extremely **likely** (1)
- ◯ Somewhat likely (2)
- ◯ Neither likely nor unlikely (3)
- ◯ Somewhat unlikely (4)
- ◯ Extremely **unlikely** (5)
- ◯ Unable to assess as did not complete sufficient amount of course

Q How likely are you to **recommend** this course to a friend/family with MS?

- ◯ Extremely **likely** (1)
- ◯ Somewhat likely (2)
- ◯ Neither likely nor unlikely (3)
- ◯ Somewhat unlikely (4)
- ◯ Extremely **unlikely** (5)

Q How familiar were you with the content **prior** the course?

- ◯ I was familiar with all of the content
- ◯ I was familiar with most of the content
- ◯ Some of the content was new to me
- ◯ Most of the content was new to me
- ◯ All of the content was new to me

Q As **a result of taking course**, how likely are you to change the following lifestyle behaviours?

	Extremely **unlikely** (1)	Moderately unlikely (2)	Neither likely nor unlikely (3)	Moderately likely (4)	Extremely **likely** (5)	Not applicable (6)
Diet (1)	◯	◯	◯	◯	◯	◯
Smoking (2)	◯	◯	◯	◯	◯	◯
Omega-3 supplementation (3)	◯	◯	◯	◯	◯	◯
Vitamin D supplementation (4)	◯	◯	◯	◯	◯	◯
Sun exposure (5)	◯	◯	◯	◯	◯	◯
Exercise (6)	◯	◯	◯	◯	◯	◯
Meditation (7)	◯	◯	◯	◯	◯	◯
Other stress-reduction activities (8)	◯	◯	◯	◯	◯	◯

Q As **a result of taking the modules**, how likely are you to change the following lifestyle behaviours? (If you did not complete the module, select not applicable)

	Extremely **unlikely** (1)	Moderately unlikely (2)	Neither likely nor unlikely (3)	Moderately likely (4)	Extremely **likely** (5)	Not applicable (6)
Diet (1)	○	○	○	○	○	○
Smoking (2)	○	○	○	○	○	○
Omega-3 supplementation (3)	○	○	○	○	○	○
Vitamin D supplementation (4)	○	○	○	○	○	○
Sun exposure (5)	○	○	○	○	○	○
Exercise (6)	○	○	○	○	○	○
Meditation (7)	○	○	○	○	○	○
Other stress-reduction activities (8)	○	○	○	○	○	○

Q Any other lifestyle behaviours you are likely to change as a **result of taking the course**?

____

Q Which topic(s) would you have liked additional information on (you may select more than one response)?

- ☐ What is MS (1)
- ☐ Eat well (2)
- ☐ Sunlight and vitamin D (3)
- ☐ Exercise (4)
- ☐ Stress management (5)
- ☐ Medication and family prevention (6)
- ☐ Change your life, for life (7)
- ☐ Other (please specify) (8) ____
- ☐ None (9)

Q Did you participate in the community forum?

- ○ Yes
- ○ No
- ○ Could not find
- ○ Other ____

Q Did you find it useful? (please specify reasons)

- ◯ Yes ____
- ◯ No ____
- ◯ Other ____

Q Is there anything else you would like to share with us *before submitting the survey*?

____
