# Supplementary material for: A Web-Based Lifestyle-Related Course for People Living With Multiple Sclerosis: Quantitative Evaluation of Course Completion, Satisfaction, and Lifestyle Changes Among Participants Enrolled in a Randomized Controlled Trial
Source: JMIR Hum Factors. 2025 May 26;12:e59363. doi: 10.2196/59363 (PMC12149781; doi:10.2196/59363)
Supplement: Multimedia Appendix 2 [file humanfactors_v12i1e59363_app2.pptx]

## Slide 1
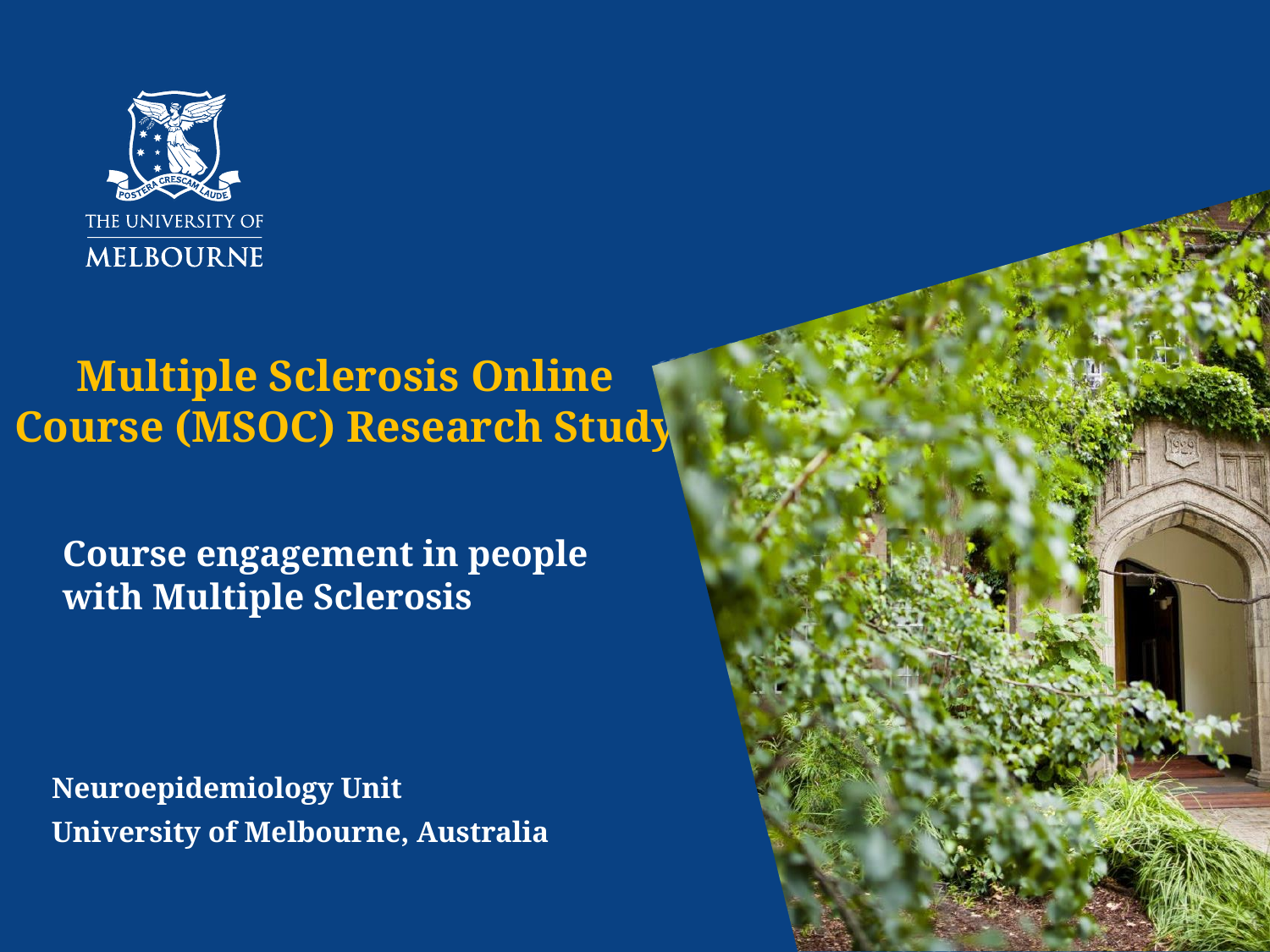

Multiple Sclerosis Online Course (MSOC) Research Study
# Course engagement in people with Multiple Sclerosis
Neuroepidemiology Unit
University of Melbourne, Australia

## Slide 2
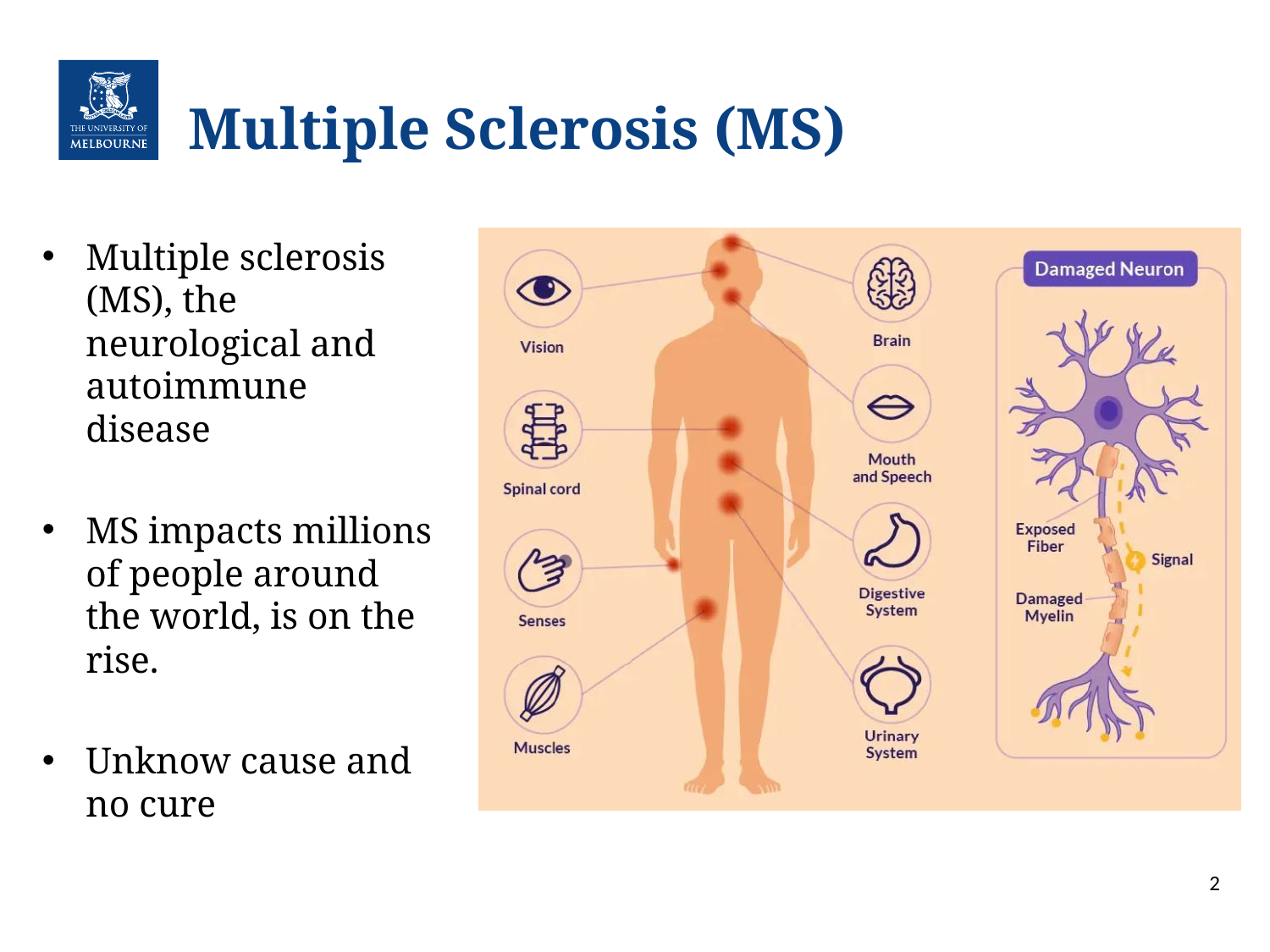

# Multiple Sclerosis (MS)
Multiple sclerosis (MS), the neurological and autoimmune disease
MS impacts millions of people around the world, is on the rise.
Unknow cause and no cure
2

## Slide 3
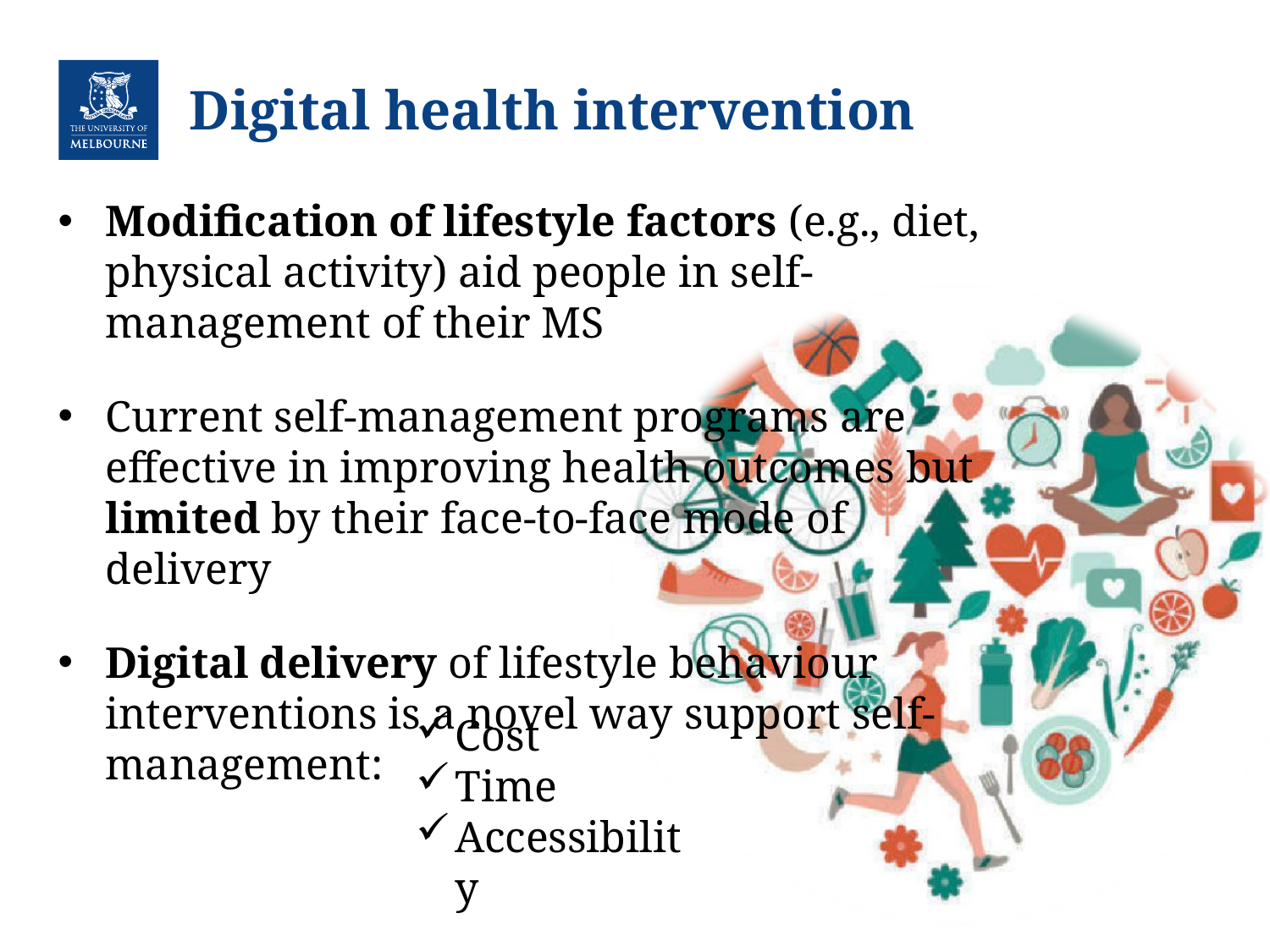

# Digital health intervention
Modification of lifestyle factors (e.g., diet, physical activity) aid people in self-management of their MS
Current self-management programs are effective in improving health outcomes but limited by their face-to-face mode of delivery
Digital delivery of lifestyle behaviour interventions is a novel way support self-management:
Cost
Time
Accessibility

## Slide 4
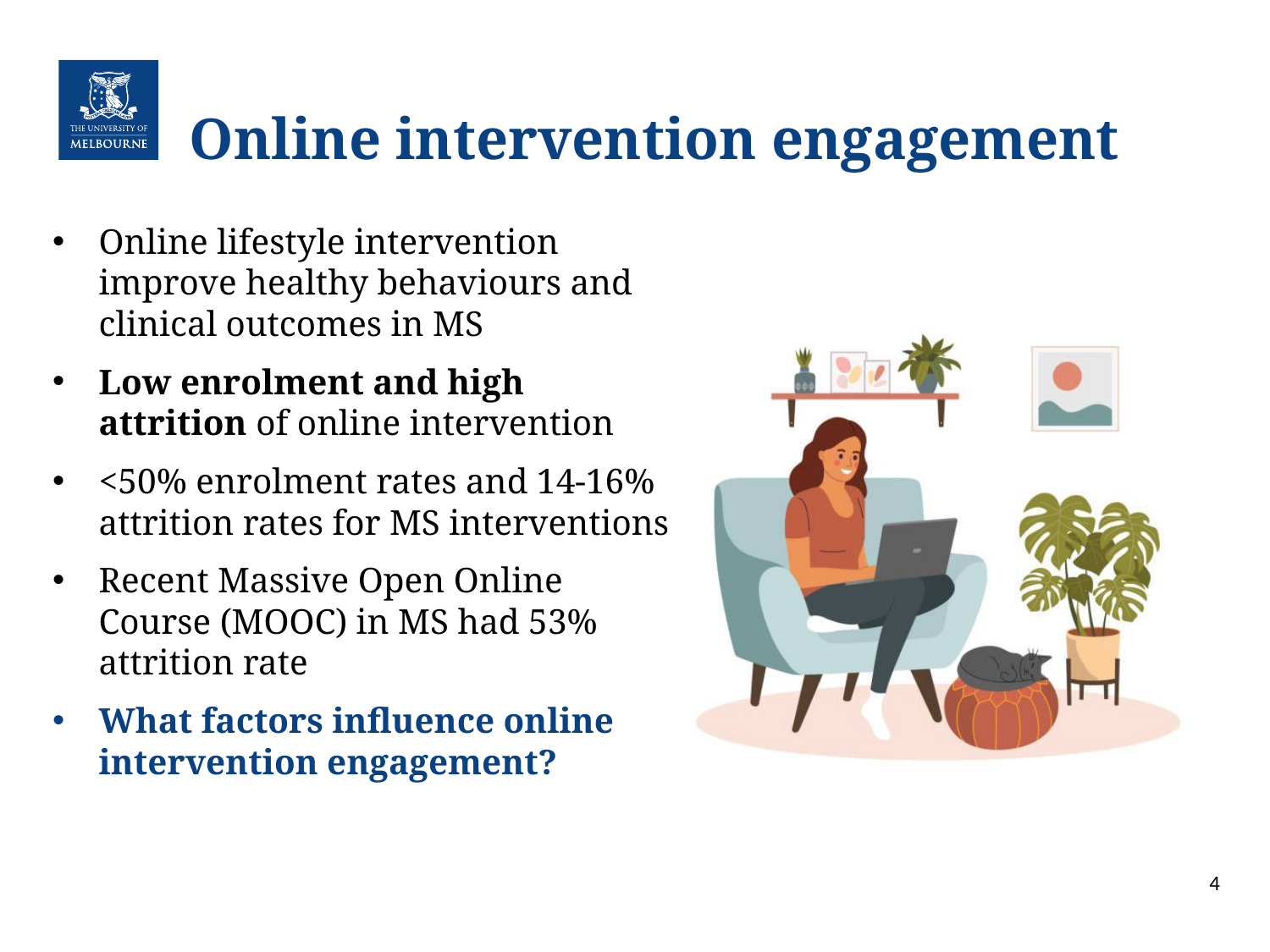

# Online intervention engagement
Online lifestyle intervention improve healthy behaviours and clinical outcomes in MS
Low enrolment and high attrition of online intervention
<50% enrolment rates and 14-16% attrition rates for MS interventions
Recent Massive Open Online Course (MOOC) in MS had 53% attrition rate
What factors influence online intervention engagement?
4

## Slide 5
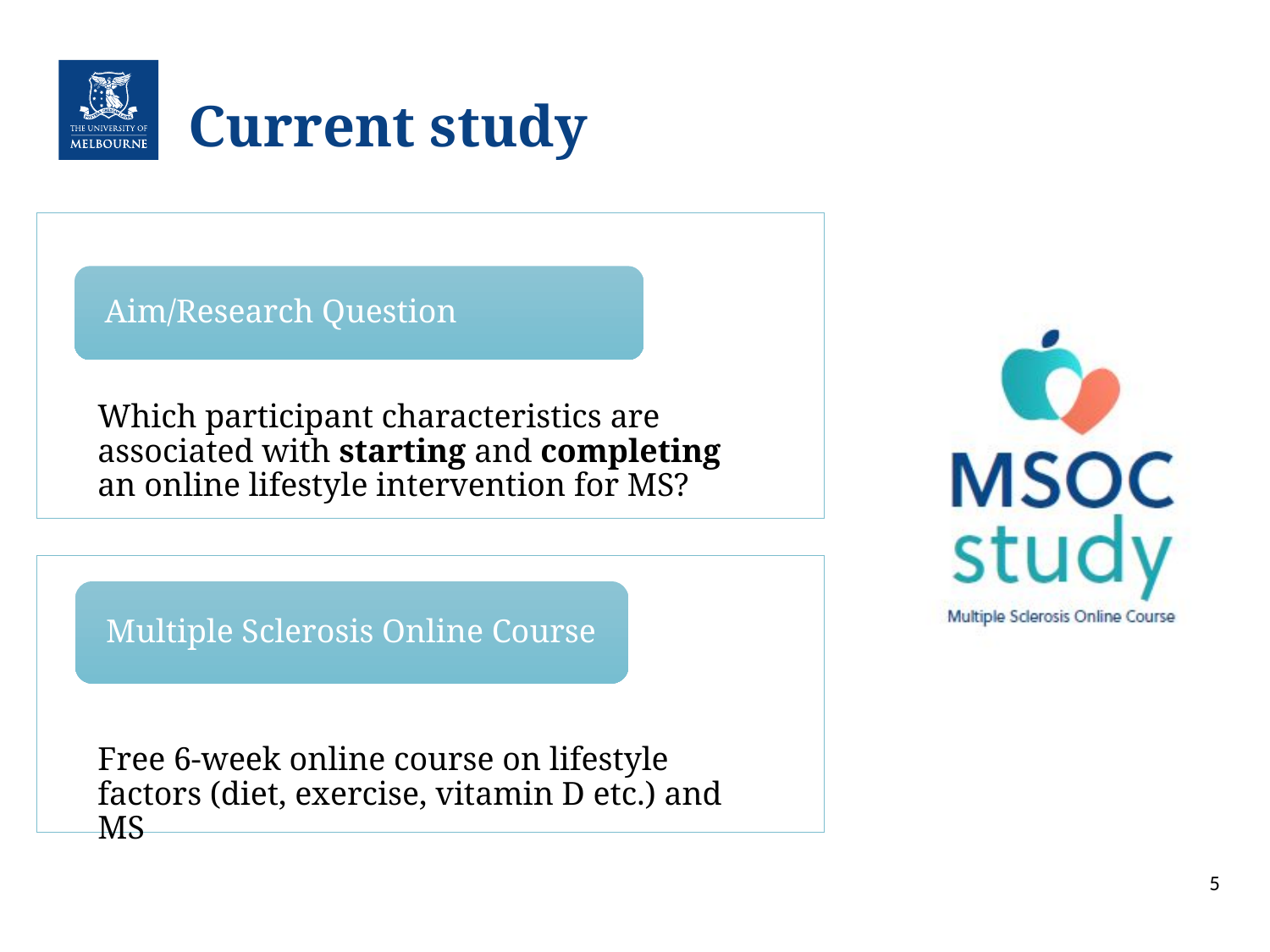

# Current study
5

## Slide 6
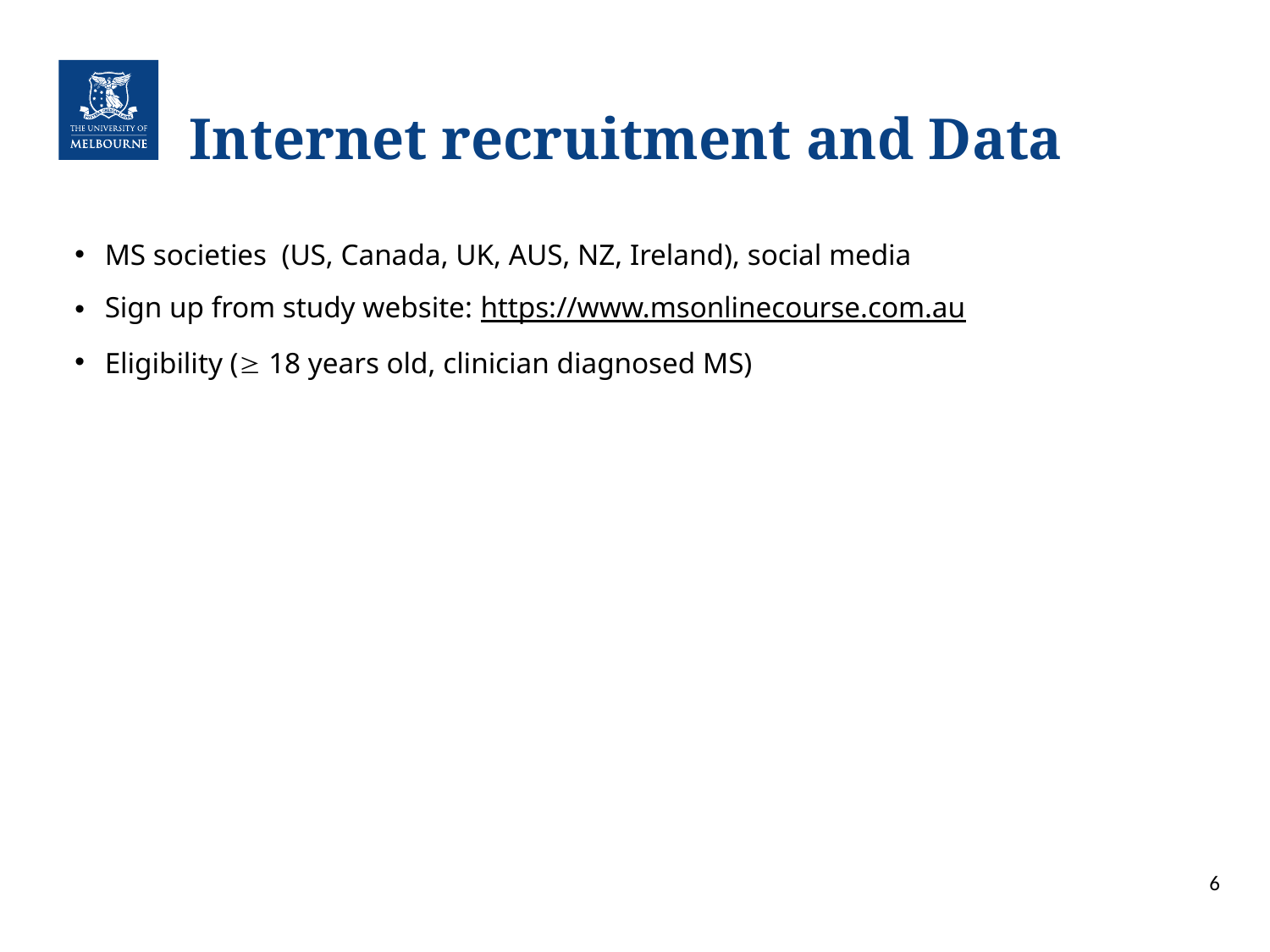

# Internet recruitment and Data
MS societies (US, Canada, UK, AUS, NZ, Ireland), social media
Sign up from study website: https://www.msonlinecourse.com.au
Eligibility (≥ 18 years old, clinician diagnosed MS)
6

## Slide 7
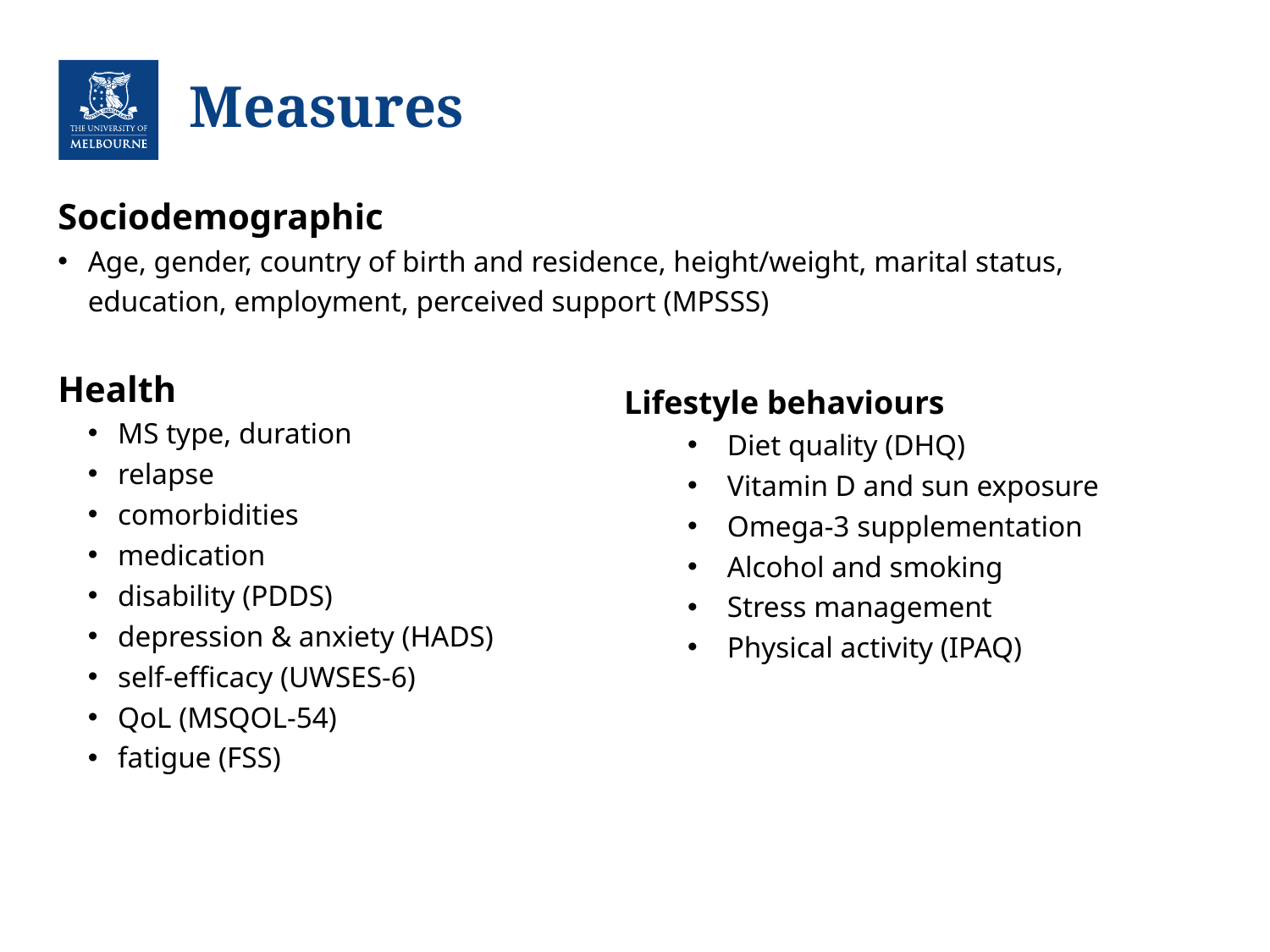

# Measures
Sociodemographic
Age, gender, country of birth and residence, height/weight, marital status, education, employment, perceived support (MPSSS)
Health
MS type, duration
relapse
comorbidities
medication
disability (PDDS)
depression & anxiety (HADS)
self-efficacy (UWSES-6)
QoL (MSQOL-54)
fatigue (FSS)
Lifestyle behaviours
Diet quality (DHQ)
Vitamin D and sun exposure
Omega-3 supplementation
Alcohol and smoking
Stress management
Physical activity (IPAQ)

## Slide 8
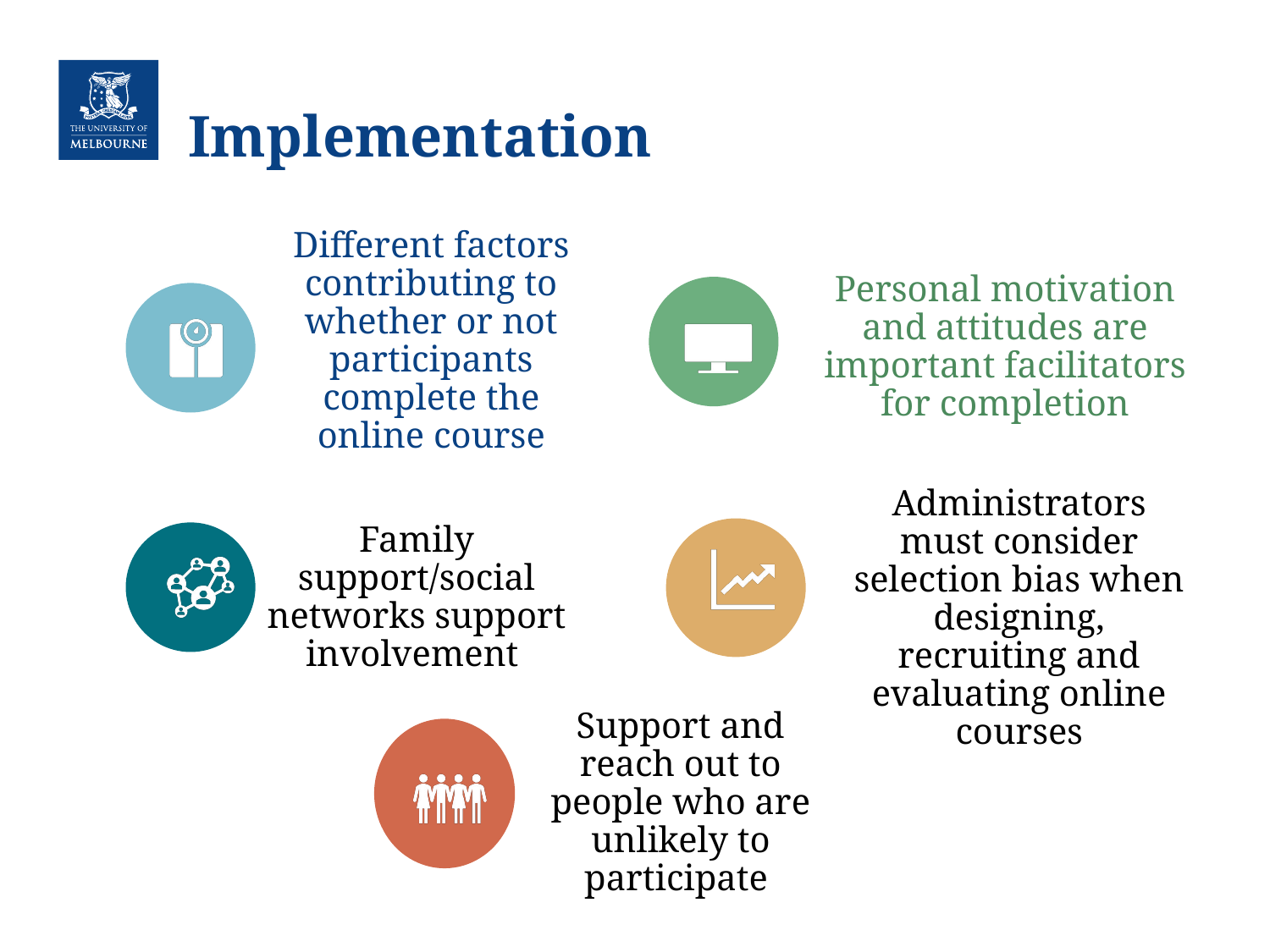

Implementation
# Re-development for MSOC study

## Slide 9
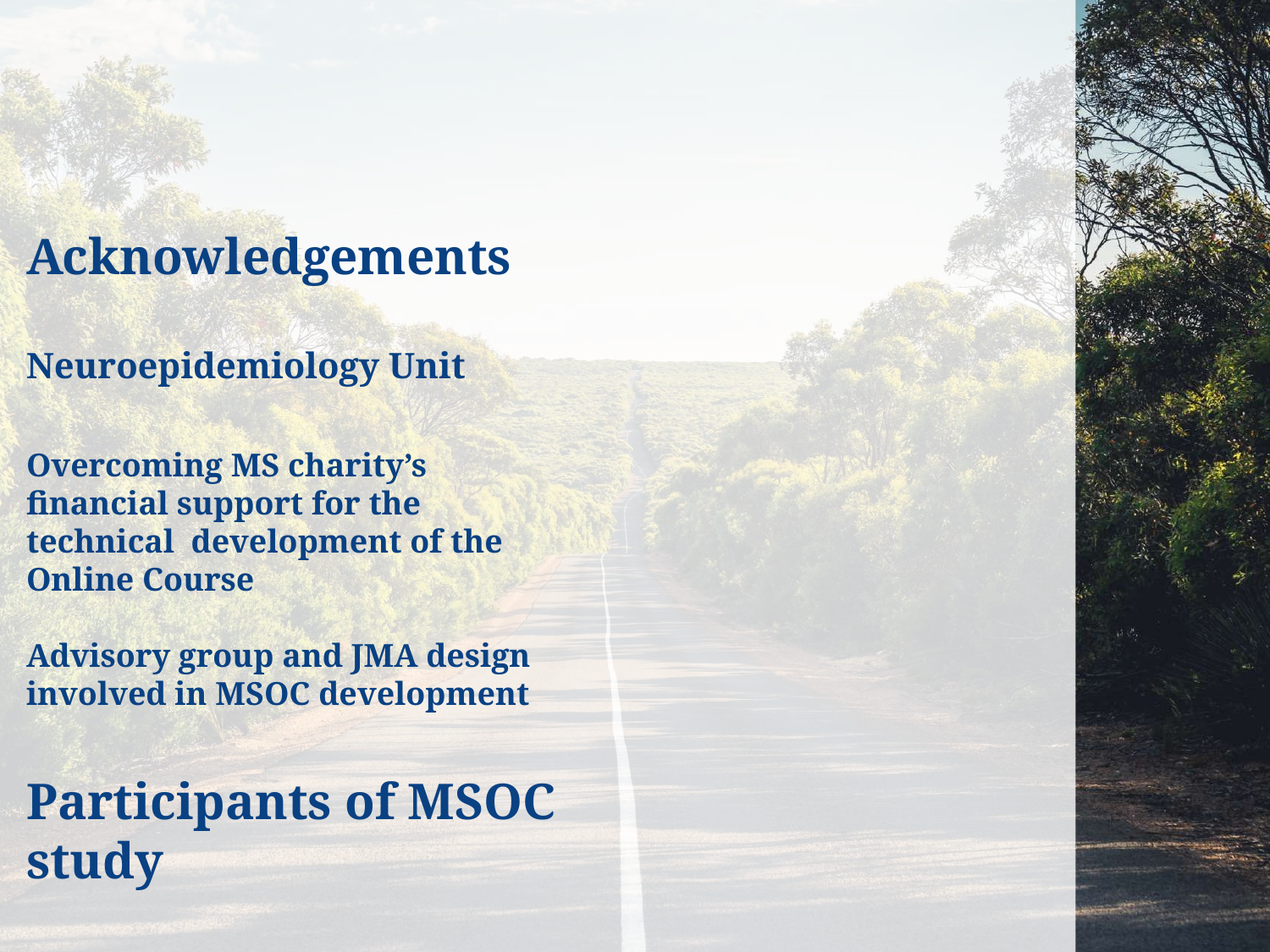

# Acknowledgements Neuroepidemiology Unit Overcoming MS charity’s financial support for the technical development of the Online Course Advisory group and JMA design involved in MSOC development Participants of MSOC study

## Slide 10
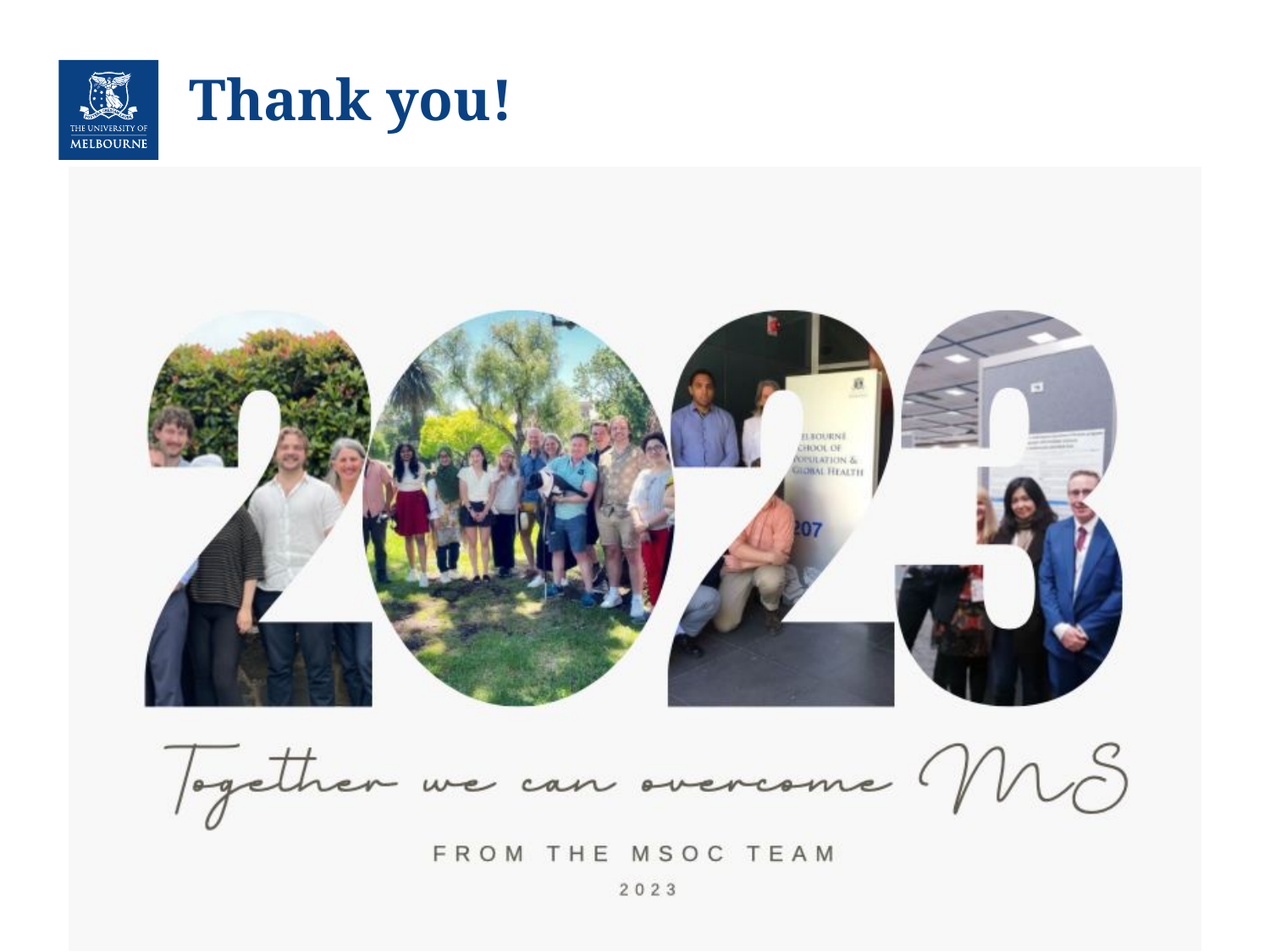

# Thank you!
