# Supplementary figures and images for: A Web-Based Lifestyle-Related Course for People Living With Multiple Sclerosis: Quantitative Evaluation of Course Completion, Satisfaction, and Lifestyle Changes Among Participants Enrolled in a Randomized Controlled Trial
Source: JMIR Hum Factors. 2025 May 26;12:e59363. doi: 10.2196/59363 (PMC12149781; doi:10.2196/59363)

## Slide 1
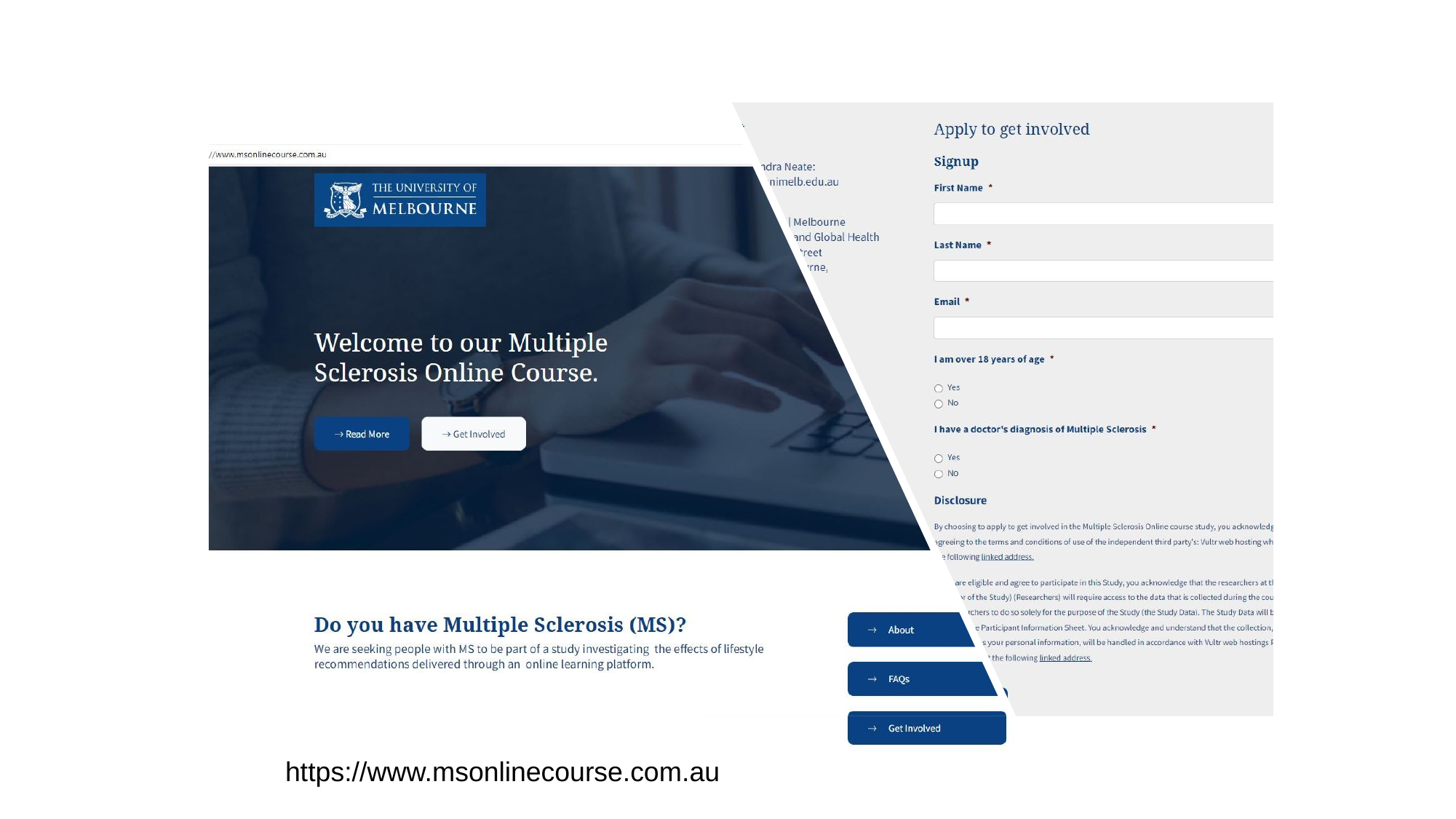

https://www.msonlinecourse.com.au

Supplement: Multimedia Appendix 3 [file humanfactors_v12i1e59363_app3.pptx]
