# Supplementary material for: A Web-Based Lifestyle-Related Course for People Living With Multiple Sclerosis: Quantitative Evaluation of Course Completion, Satisfaction, and Lifestyle Changes Among Participants Enrolled in a Randomized Controlled Trial
Source: JMIR Hum Factors. 2025 May 26;12:e59363. doi: 10.2196/59363 (PMC12149781; doi:10.2196/59363)
Supplement: Multimedia Appendix 4 [file humanfactors_v12i1e59363_app4.docx]

**Supplementary Table 1.** Summary of recommendations of the IC and SCC arms.

| Week | Modules | IC^a^ recommendations | SCC^b^ recommendations |
| --- | --- | --- | --- |
| 1 | 1. Introduction to the course | Welcome to the course, including how to proceed, and navigate the course modules. An overview of MS^c^ will also be provided. | Welcome to the course, including how to proceed, how to navigate, what to expect, and to outline the endpoints for participants. An overview of MS will also be provided. |
|  | 1. Eat well | Evidence behind the role of diet in MS risk, disease activity, and QoL^d^, including saturated fat and its relationships with MS onset and progression. Recommends a plant-based wholefood diet plus seafood, with <20 g/day saturated fat, as well as omega-3 fatty acid supplement use, or 20-40 ml of flaxseed oil (or equivalent) per day. | Information regarding the importance of a balanced diet. Public Health England Eatwell guide presented. Information from the US National MS society presenting a selection of diets used by people with MS including gluten free, Paleolithic diet, McDougal diet, Mediterranean diet and Swank diet. Alcohol consumption should follow national guidelines. |
| 2 | 1. Sunlight and vitamin D | Detailed information about how vitamin D is made from skin exposure to sunlight, along with evidence supporting the potential role of vitamin D in MS risk and progression Recommendations for optimal levels of vitamin D supplementation and blood levels are presented. Recommends sun exposure of at least 15 minutes per day, 3-5 times a week, and vitamin D3 supplement use of at least 5,000 IU per day. | Information regarding Vitamin D and MS development, latitude gradient of MS and how the body produce vitamin D from sunlight. No specific recommendations on sun exposure or supplementation described. Three options presented: wait until more information is available, supplement ‘blindly’ or supplement if blood 25-hydroxyvitamin D levels are low. |
|  | 1. Exercise | The benefits of exercise (neurological, cognitive, physical), how and why to implement an exercise plan with video examples provided. Recommends 20-30 minutes, 5 times/week exercise outdoor preferably. | Presented the critical role exercise plays in MS. Recommend 30 minutes or more of moderate aerobic activity and strength training at least twice per week |
| 3 | 1. Meditation and use the mind-body connection | Introduction to mental health and relevance in MS, to the science behind stress and its link to inflammation/MS. Mind-body connection, meditation, and other stress reduction techniques to supplement the practical sessions on meditation are delivered along with how to develop a mental health and wellbeing improvement strategy. Recommends 30 minutes or more of daily meditation. | No conclusive link between stress and MS, as the evidence to date is contradictory. Introduction to the associations between MS and chronic distress and stressful life events. |
|  | 1. Medication and family prevention | Genetic risk of getting MS and prevention via lifestyle. Risks associated with smoking and excessive alcohol intake and MS progression. Role of medication in MS discussed. Recommends no tobacco smoking and avoid passive smoke exposure, and moderate alcohol intake. | Genetic risk of getting MS. Smoking increases the risk of MS. The role of medication in MS discussed. |
| 4 | 1. Review and consolidation (Change your life, for life) | Program overview and recap, and an outline of next steps to take. Follow-up questionnaires for longitudinal follow-up discussed and participants asked to complete, and link to forums for further engagement (aimed at enhancing retention).  Concluding remarks and closing ceremony. | Program overview and recap. Follow-up questionnaires for longitudinal follow-up discussed and participants asked to complete, and link to forums for further engagement (aimed at enhancing retention). |
| 5/6 | Catch-up | Participants have 2 weeks to complete any modules missed. | Participants have 2 weeks to complete any modules missed. |

^a^ SCC: standard care course.

^b^ IC: intervention course.

^c^ MS: multiple sclerosis

^d^ QoL: quality of life

**Supplemental Table 2** Country of residence the study sample (N = 291).

|  | N | % |
| --- | --- | --- |
|  |  |  |
| **Country residence** |  |  |
| Australia | 73 | 25.2 |
| Austria | 1 | 0.3 |
| Belgium | 1 | 0.3 |
| Bosnia and Herzegovina | 1 | 0.3 |
| Bulgaria | 7 | 2.4 |
| Canada | 46 | 15.9 |
| Croatia | 2 | 0.7 |
| Cyprus | 1 | 0.3 |
| Czech Republic | 3 | 1.0 |
| Estonia | 4 | 1.4 |
| Finland | 1 | 0.3 |
| France | 4 | 1.4 |
| Germany | 3 | 1.0 |
| Iran | 1 | 0.3 |
| Ireland | 4 | 1.4 |
| Israel | 1 | 0.3 |
| Italy | 3 | 1.0 |
| Mexico | 2 | 0.7 |
| Netherlands | 2 | 0.7 |
| New Zealand | 12 | 4.1 |
| Norway | 1 | 0.3 |
| Portugal | 1 | 0.3 |
| Russian Federation | 3 | 1.0 |
| Serbia | 3 | 1.0 |
| South Africa | 3 | 1.0 |
| Spain | 2 | 0.7 |
| Sweden | 2 | 0.7 |
| Switzerland | 1 | 0.3 |
| Taiwan | 1 | 0.3 |
| Ukraine | 3 | 1.0 |
| United Kingdom | 40 | 13.8 |
| United States | 58 | 20.0 |
| Missing | 1 | 0.3 |
| **Total** | **290** | **100.0** |

**Supplementary Table 3**. Extended responses to the usefulness of community forums.

|  | Yes, useful | No, not useful |
| --- | --- | --- |
|  |  |  |
| **Responses** | Helpful to see other’s comments | Posted but never got responses |
|  | Nice to see others have same concerns I do | There were more individual comments |
|  | Great resources | The engagement of others was lower than expected. |
|  | Exchange of experiences | Not a lot of other people there to interact with - no critical mass. |
|  | Easy to share experiences | Not my thing |
|  | Responses to comments were helpful. | It was not well structured or formatted. I could not reply to any comment. |
|  | Shared views | No reason |
|  | Nice to not feel I am on my own with my MS | Not a lot of comments |
|  | I asked questions when there was something a did not understand. | Nothing new. |
|  | Answers to specific questions raised by the course | My questions weren’t all answered and little other activity in the forum |
|  | It was great to learn about other people with MS around the world. | Not as much engagement as i would have hoped |
|  | Sharing experiences | Did not like the responses |
|  | It was interesting to hear others’ perspectives. | I asked a question, and no one answered |
|  | There wasn't a lot of interaction but was interesting to read what others are asking or experiencing, and t.. | There wasn’t really a lot of back and forth and no neared comments so hard to respond |
|  | Other people's advice |  |
|  | It’s useful to find out more about topics |  |
|  | Somewhat useful; I couldn’t open for all the conversations |  |
|  | Sharing experience |  |
|  | Hearing from others and sharing own insights gained. |  |
|  | Nice to see others’ comments |  |
|  | It was good to read about other people's experiences, struggles and successes. |  |
|  | Knowing the issues are not just 'yours' |  |
|  | Somewhat. Questions I had were often already answered in the other participants questions |  |
|  | My question was answered |  |
|  | Interacting with others is very useful |  |
|  | To read other comments |  |
|  | I appreciated that there were responses and resources in the forum |  |
|  | I found it really useful both to view others’ perspectives and ideas and to receive expert advice from facilitators. |  |
|  | I enjoyed reading other people's experiences |  |
|  | It’s always helpful to hear how others cope. It’s also very helpful to hear suggestions. |  |
|  | I had some questions on differences in terminology which were resolved |  |
|  | Useful to see how other people were getting on |  |
|  | Hearing others’ ideas/experiences |  |
|  | In reading others' posts, I realized I was not alone in many aspects. |  |
|  | Helpful to see others’ comments but I did hesitate because I am not fully “out”. |  |
|  | They helped me understand some doubts I had about the information in the course. |  |
|  | Hear other people's thoughts and opinions. |  |
|  | Easy and no stressful |  |
|  | To learn of other people's experiences |  |
|  | Always good to hear from others with MS and their take on things |  |
|  | Moderately reading other experiences |  |
|  | Good to have feedback and responses to questions. |  |
|  | Enjoyed recording the graduation |  |
|  | I felt like I was with a tribe and not alone! |  |
|  | It was right at the bottom and couldn’t miss it. |  |

Note: Participants who answered 'yes' to participating in the community forums were subsequently asked if they found them useful and extend their responses.
